# Exosome-Based Drug Delivery: A Next-Generation Platform for Cancer, Infection, Neurological and Immunological Diseases, Gene Therapy and Regenerative Medicine

**DOI:** 10.3390/pharmaceutics17101336

**Published:** 2025-10-15

**Authors:** Dolores R. Serrano, Francisco Juste, Brayan J. Anaya, Bianca I. Ramirez, Sergio A. Sánchez-Guirales, John M. Quispillo, Ester M. Hernandez, Jesus A. Simon, Jose M. Trallero, Celia Serrano, Satyavati Rawat, Aikaterini Lalatsa

**Affiliations:** 1 Department of Pharmaceutics and Food Science, School of Pharmacy, Complutense University of Madrid, 28040 Madrid, Spain; branaya@ucm.es (B.J.A.); sergsa16@ucm.es (S.A.S.-G.); johnmqui@ucm.es (J.M.Q.);; 2 Instituto Universitario de Farmacia Industrial, Universidad Complutense de Madrid, 28040 Madrid, Spain; 3 Laboratorios Juste, San Pablo 27, Coslada, 28820 Madrid, Spain; 4 Escuela Superior Politécnica de Chimborazo, Universidad en Riobamba, Riobamba 060155, Ecuador; 5 Department of Botany, Kurukshetra University, Thanesar 136119, India; 6 Formulation Unit, Institute of Pharmacy and Biomedical Sciences, University of Strathclyde, 161 Cathedral Street, Glasgow G4 0RE, UK

**Keywords:** exosomes, tissue regeneration, cancer, infection, drug delivery, CRISPR-Cas9, extracellular vesicles, cosmeceuticals

## Abstract

Exosomes, naturally derived extracellular vesicles, have emerged as powerful bio-nanocarriers in precision medicine. Their endogenous origin, biocompatibility, and ability to encapsulate and deliver diverse therapeutic payloads position them as transformative tools in drug delivery, gene therapy, and regenerative medicine. This review presents a comprehensive analysis of exosome-based therapeutics across multiple biomedical domains, including cancer, neurological and infectious diseases, immune modulation, and tissue repair. Exosomes derived from stem cells, immune cells, or engineered lines can be loaded with small molecules, RNA, or CRISPR-Cas systems, offering highly specific and low-immunogenic alternatives to viral vectors or synthetic nanoparticles. We explore endogenous and exogenous loading strategies, surface functionalization techniques for targeted delivery, and innovations that allow exosomes to traverse physiological barriers such as the blood–brain barrier. Furthermore, exosomes demonstrate immunomodulatory and regenerative properties in autoimmune and degenerative conditions, with promising roles in skin rejuvenation and cosmeceuticals. Despite their potential, challenges remain in large-scale production, cargo loading efficiency, and regulatory translation. Recent clinical trials and industry efforts underscore the accelerating momentum in this field. Exosomes represent a promising platform in precision medicine, though further standardization and validation are required before widespread clinical use. This review offers critical insights into current technologies, therapeutic mechanisms, and future directions to unlock the full translational potential of exosomes in clinical practice.

## 1. Introduction

The development of effective and precise drug delivery systems remains one of the most pressing challenges in modern medicine [1]. Despite significant progress in pharmaceutical research, conventional drug delivery methods including systemic administration of small-molecule drugs, liposomes, polymeric nanoparticles, and micelles often face several limitations. These include poor solubility of drugs, rapid clearance from the bloodstream, lack of specificity toward target tissues, off-target effects, dose-limiting toxicities, and induction of immune responses. These obstacles are especially pronounced in the treatment of complex diseases such as cancer, chronic infections, and degenerative conditions, where therapeutic efficacy requires not only the delivery of active agents but also their sustained and controlled release at the site of action.

Over the past decade, exosomes have emerged as a highly promising platform for overcoming these limitations. Exosomes are a subtype of extracellular vesicles (EVs), typically ranging from 30 nm to 150 nm in diameter, and are naturally secreted by a wide variety of cell types. They originate from the inward budding of multivesicular bodies and are released into the extracellular space upon fusion with the plasma membrane. Exosomes carry a rich cargo of biologically active molecules, including proteins, lipids, RNAs (such as mRNAs and microRNAs), and metabolites, which they can transfer to recipient cells. Through this intercellular communication mechanism, exosomes play critical roles in regulating physiological and pathological processes, including immune responses, tissue regeneration, and disease progression [2].

One of the most attractive features of exosomes as drug delivery vehicles lies in their natural origin and inherent biocompatibility [3]. Being derived from the body’s own cells, exosomes are typically well-tolerated by the immune system, reducing the risk of immunogenicity often associated with synthetic delivery systems. In addition, exosomes possess intrinsic targeting capabilities, with surface proteins such as tetraspanins (CD9, CD63, CD81), integrins, and other adhesion molecules that can influence biodistribution and cell-specific uptake. These features provide a foundation for the development of personalized, cell-derived therapeutics with reduced systemic toxicity and improved pharmacokinetics [4].

Moreover, exosomes can be engineered to enhance their therapeutic potential. Techniques such as electroporation, sonication, extrusion, and chemical conjugation can be employed to load exosomes with various therapeutic agents, including chemotherapeutic drugs, siRNA, miRNA, proteins, or CRISPR/Cas9 components. In parallel, surface modifications can be used to improve targeting or circulation time. Importantly, exosomes can also be produced from specific donor cells, such as mesenchymal stem cells (MSCs), immune cells, or cancer cells, each imparting unique biological properties that may enhance therapeutic effectiveness depending on the disease context [5].

Given these capabilities, exosome-based drug delivery systems are gaining significant attention in three major biomedical domains: oncology, regenerative medicine, and infectious disease treatment. In cancer therapy, exosomes offer the ability to deliver cytotoxic agents or gene therapies directly to tumor sites, bypassing some of the resistance mechanisms associated with conventional therapies [6]. In regenerative medicine, stem-cell-derived exosomes have shown potential in promoting tissue repair, angiogenesis, and anti-inflammatory responses, offering a cell-free alternative to stem cell transplantation [7]. In the context of infectious diseases, exosomes may serve both as therapeutic agents by delivering antimicrobial drugs or immune modulators and as diagnostic biomarkers, given their ability to reflect the physiological status of their parent cells [8].

Exosomes can be viewed not only as vesicular carriers but also through the lens of compartmentalization principles—in a similar conceptual space to biomolecular coacervates, liposomes, and phase-separated microdroplets. Like coacervates, exosomes feature selective partitioning of biomolecules: specific RNAs, proteins, or lipids are enriched inside relative to the surrounding cytosol or extracellular milieu. The mechanisms underlying this enrichment (e.g., affinity interactions, electrostatics, binding motifs, membrane microdomains) share conceptual parallels with driven phase separation in coacervate droplets. Moreover, exosomes exhibit responsive behavior (e.g., cargo release triggered by pH, redox, or enzyme cues) akin to stimuli-responsive coacervates. Exploring these analogies helps us think more broadly about cargo sorting, stability, and triggerable release in therapeutic exosome engineering [9,10].

Despite the promising outlook, the clinical translation of exosome-based therapies still faces several challenges. Standardization of isolation, purification, and characterization methods remains an ongoing issue, with various techniques yielding exosomes of different purity, size, and functionality [11]. There is also a need for scalable and reproducible manufacturing processes that comply with regulatory standards for clinical-grade materials [12]. Furthermore, questions remain about pharmacokinetics, biodistribution, and long-term safety of exosome-based therapeutics. Addressing these challenges will be essential to realizing the full potential of exosomes in clinical applications.

In this review, we aim to provide a comprehensive overview of the current state and prospects of exosome-based drug delivery systems, with a focus on their applications in cancer, regenerative medicine, and infectious diseases. Biogenesis, composition, and isolation methods of exosomes will be covered, followed by different strategies for drug loading and engineering. We then explore specific therapeutic applications in each of the three focal areas, highlighting preclinical and clinical findings, as well as the mechanisms by which exosomes exert their therapeutic effects. Finally, we discuss the translational hurdles that must be addressed and propose future directions for advancing the field.

## 2. Biology and Biogenesis of Exosomes

### 2.1. Definition and Classification of EVs

EVs refers to a heterogeneous population of membrane-bound particles secreted by cells, encompassing subtypes such as exosomes, microvesicles, and apoptotic bodies (each differing by biogenesis, size, and cargo) [13]. The consensus in the field is that the term exosome should be reserved for EVs of endosomal origin, specifically those formed inside multivesicular bodies (MVBs) and released by exocytosis, typically between 30 nm and 150 nm in size (sometimes expanded to ~200 nm in broader definitions). In contrast, microvesicles (also called ectosomes) arise via direct budding from the plasma membrane (size ~100–1000 nm), and apoptotic bodies are released from dying cells during programmed cell death (>1 µm) [14].

EV classification may also consider characteristics such as density, biomolecular profile, or cellular origin. However, due to overlapping properties and measurement limitations, strict biochemical or biogenetic verification is often lacking in such cases the broader term “EV” is preferred unless clear origin is demonstrated [14]. For the purposes of this review, “exosomes” will refer to EVs of endosomal origin, recognizing that strict separation is experimentally challenging.

### 2.2. Mechanisms of Exosome Formation (Endosomal Pathway)

#### 2.2.1. Intraluminal Vesicle (ILV) Formation Within MVBs

Exosome biogenesis begins with the inward budding of the endosomal membrane, creating intraluminal vesicles (ILVs) within late endosomes or MVBs. Cargo sorting into these ILVs can occur via the classic endosomal sorting complex required for transport (ESCRT-dependent pathway), involving the sequential action of ESCRT-0, -I, -II, and -III complexes, along with associated ATPase VPS4 for membrane scission. ESCRT-0 initially binds ubiquitinated proteins and clusters them; ESCRT-I/II drive membrane deformation; ESCRT-III mediates membrane neck formation and constriction, ultimately leading to vesicle budding. VPS4-mediated disassembly completes the process [15]. This is broadly conserved and operates in many cell types including epithelial, immune, and tumor cells. Experimental depletion of ESCRT components such as TSG101, ALIX, or VPS4A markedly reduces exosome secretion, confirming their central role in vesicle biogenesis. Functionally, this pathway tends to produce exosomes enriched in ubiquitinated proteins, RNA-binding proteins, and specific miRNAs—cargo profiles that are often more uniform and easier to manipulate for therapeutic engineering [15].

In contrast, ESCRT-independent pathways rely on lipid microdomains and tetraspanin-rich membranes. One key mechanism involves neutral sphingomyelinase 2 (nSMase2), which generates ceramide, a bioactive lipid that induces negative membrane curvature and facilitates budding of ILVs in the absence of ESCRT machinery. Tetraspanins such as CD9, CD63, and CD81 also participate by organizing microdomains that promote selective cargo clustering and vesicle formation. These lipid-driven routes are particularly active in fibroblasts, certain neuronal and tumor cells, and may favor inclusion of signaling lipids and specific proteins rather than ubiquitinated cargo (Table 1).

The relative contribution of ESCRT-dependent versus ESCRT-independent mechanisms varies among cell types and physiological conditions. For example, in dendritic cells, inhibition of nSMase2 reduces ceramide-dependent vesicle formation, whereas TSG101 knockdown primarily affects ESCRT-dependent sorting of MHC-II-containing vesicles. Such diversity implies that different donor cells may naturally bias exosome composition, influencing their downstream biological effects and suitability for therapeutic applications. Understanding these mechanistic differences is therefore essential for rational design of engineered exosomes, optimization of yield, and control of cargo composition in clinical manufacturing [15,16].

#### 2.2.2. MVB Fate: Secretion vs. Degradation

**Figure 1:**
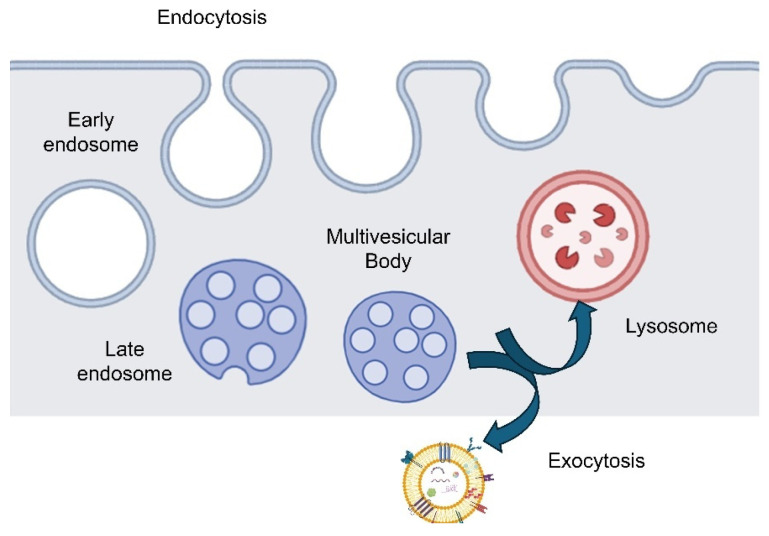
MVB Fate: Secretion vs. Degradation. Schematic illustration of the endosomal maturation pathway showing how multivesicular bodies (MVBs) can follow two alternative fates: (i) fusion with the plasma membrane, leading to the secretion of exosomes into the extracellular space, or (ii) fusion with lysosomes, resulting in degradation of intraluminal vesicles.

Once ILVs have formed, MVBs face two main fates: fusion with the plasma membrane to release ILVs as exosomes, or fusion with lysosomes/autophagosomes for degradation (Figure 1). Factors such as the composition of cargo, MVB-associated proteins, and cellular signals influence routing. Notably, different cargo types may bias MVBs toward the secretory pathway, creating heterogeneity among released exosomes and reflecting functional specialization [16].

#### 2.2.3. Regulation and Heterogeneity

Multiple pathways can operate simultaneously or sequentially within a single cell, leading to diverse subpopulations of exosomes even from the same donor. Regulatory mechanisms depend heavily on cell type, activation state, and cellular stresses; thus, the exosome output is dynamic and context-dependent [16].

### 2.3. Surface Markers and Molecular Cargo

#### 2.3.1. Common Surface Markers

**Figure 2:**
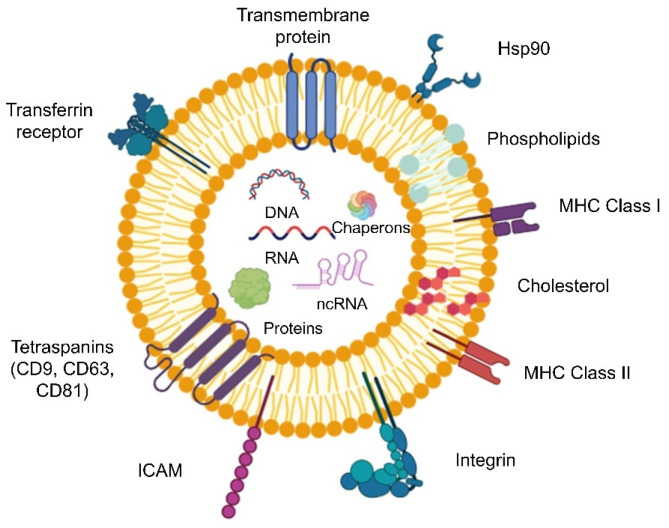
Schematic representation of exosome composition. Diagram of the typical molecular components of exosomes, including lipid bilayer structure enriched with cholesterol, sphingomyelin, and ceramide, surface proteins such as tetraspanins (CD9, CD63, CD81), integrins, adhesion molecules, and enclosed cargos such as nucleic acids (mRNA, miRNA, siRNA), proteins (heat shock proteins, signaling molecules), and metabolites.

Exosomes are enriched in tetraspanins such as CD9, CD63, and CD81, widely used as canonical exosome markers and critical for vesicle formation, cargo selection, and target-cell adhesion. Additional surface proteins include heat-shock proteins (Hsp70, Hsp90), integrins, adhesion molecules, receptors (e.g., MHC molecules in immune contexts, transferrin, ICAM, primary entry receptor for Coxsackievirus A21), and other membrane-associated components depending on the cell of origin (Figure 2) [17].

#### 2.3.2. Internal Cargo: RNA, DNA, Proteins, Lipids

Exosomal cargo comprises a rich repertoire of biomolecules (Figure 2): (i) Proteins: evolutionarily conserved proteins including cytoskeletal elements (actin, tubulin), chaperon proteins, signaling molecules, metabolic enzymes, and membrane transport/fusion proteins. The exact profile is cell- and context-specific. (ii) Nucleic Acids: mRNAs, microRNAs (miRNAs) and non-coding RNA (ncRNA) are abundant in exosomes, and in some cases double-stranded DNA has also been detected. These RNAs can be delivered to recipient cells and translated or exert gene-regulatory activities. Packaging of miRNAs may follow sequence-specific motifs (e.g., GGAG EXOmotif recognized by sumoylated hnRNPA2B1), suggesting active sorting rather than passive loading. (iii) Lipids: exosomes are lipid-rich, especially in cholesterol, sphingomyelin, ceramides, saturated phosphatidylcholine, and phosphatidylethanolamine lipid composition distinct from whole-cell membranes. These lipids not only provide structural integrity but may also contribute to vesicle formation via membrane curvature and sorting [18]. This diverse cargo reflects the physiological condition of the parent cell and can be selectively enriched, making exosomes biologically informative and functionally potent.

### 2.4. Natural Role in Intercellular Communication

#### 2.4.1. Modes of Interaction

Exosomes mediate intercellular signaling through three primary modes: (i) Direct membrane fusion or endocytosis by recipients, delivering cargo internally, (ii) Receptor-mediated binding, where surface markers interact with receptors on recipient cells, and (iii) Paracrine or endocrine transport, allowing exosomes to travel via bodily fluids and act at distant sites [19,20]. These mechanisms underline the versatility of exosomes as vehicles for information exchange, capable of modulating physiological functions across tissues.

#### 2.4.2. Physiological Functions

In normal physiology, exosomes play key roles in immune modulation, tissue development, homeostasis, and waste disposal. For example, in the hematopoietic system, exosomes regulate antigen presentation and communication between dendritic cells and B cells; in the reproductive context, embryo–maternal signaling via exosomes contributes to implantation and pregnancy maintenance [20]. Exosomal RNA and protein signals also help maintain tissue health, mediate oxidative stress responses, and coordinate regenerative processes.

#### 2.4.3. Pathophysiological Roles and Disease Implications

In cancer, tumor cell-derived exosomes deliver oncogenic proteins, mutated receptors (e.g., EGFRvIII), and miRNAs to other cancer or stromal cells promoting angiogenesis, invasion, immune evasion, and organotropism. Integrins and other surface molecules on exosomes can direct metastasis to specific organs (“organ-tropic” patterns) [21].

In infection, host or pathogen-infected cell-derived exosomes can carry microbial antigens, inflammatory mediators (e.g., LPS, cytokines), or pathogen-derived RNAs, modulating immune responses in target cells or tissues [20].

In systemic disease, exosomes contribute to aging and inflammation; for example, senescent cells release EVs enriched in specific miRNAs that drive inflammatory signaling in recipient cells, contributing to tissue deterioration and age-related pathologies [13].

#### 2.4.4. Regulatory and Translational Insights

Recognition of exosomes as major mediators of intercellular crosstalk has transformed thinking into cell biology and disease models. EVs are now recognized as potential biomarkers, reflecting disease states through their cargo and as biological vehicles that can be harnessed for therapy. Understandably, this awareness has stimulated interest in bioengineering exosomes for targeted drug delivery, since their natural functions suggest advantages in stability, targeting, and biocompatibility [22].

In summary, exosomes are a distinct EV subtype defined by their endosomal origin, small size (~30 nm–150 nm), and specific cargo profile. Their biogenesis involves ILV formation within MVBs, regulated by both ESCRT-dependent and -independent pathways. Exosomes are enriched in conserved surface markers notably tetraspanins and carry diverse molecular cargo including proteins, nucleic acids, and lipids, often selectively sorted. Functionally, exosomes serve as versatile mediators of intercellular communication, influencing physiological homeostasis, immunity, development, and disease progression including cancer and infection. Understanding these fundamental biological properties lays the groundwork for exploiting exosomes as natural, programmable drug delivery vehicles in therapeutic applications.

#### 2.4.5. Immunogenicity of Exosomes

While exosomes are often regarded as low immunogenic vehicles (i.e., they themselves do not provoke strong innate or adaptive immune rejection), tumor-derived exosomes can nonetheless mediate active immunosuppression via their cargo. The distinction is that “low immunogenicity” refers to the absence of strong immune responses against the vesicle itself, whereas the biological content may modulate immune responses.

Indeed, tumor exosomes often carry immunosuppressive molecules such as PD-L1, TGF-β, FasL, miRNAs, and others, which can inhibit T-cell activation, promote regulatory T cells, induce apoptosis of effector cells, or polarize myeloid cells toward suppressive phenotypes (e.g., M2 macrophages) [23].

In particular, exosomal PD-L1 has been extensively studied. It binds PD-1 on T cells, induces T-cell exhaustion or apoptosis, and impairs antitumor immunity in vivo. In a mouse model, suppression of exosomal PD-L1 led to systemic antitumor responses and immunological memory, supporting its functional importance [24]. Also, in melanoma/mouse models, administration of exosomes with PD-L1 promotes tumor growth and reduces tumor-infiltrating CD8^+^ T cell [25].

On the flip side, exosomes derived from mesenchymal stem cells (MSCs) or other non-tumor sources tend to be well tolerated and have low immunogenicity, making them safer for therapeutic use. MSC-derived exosomes are well tolerated and have low immunogenicity compared to parent MSCs or viral vectors [26]. Clinically, MSC-EVs are being evaluated in immunomodulation/regeneration precisely because of this favorable immunological profile [27].

Thus, the paradox is resolved by appreciating that exosomes are not immunologically inert in a functional sense: their origin and cargo determine whether they are immunostimulatory, immunosuppressive, or neutral. For translation, this implies (i) avoiding or modifying tumor-derived exosomes as therapeutic vectors, (ii) using “benign” sources (e.g., MSCs, immune cells), and (iii) thoroughly characterizing immune effects (cytokine response, T cell activation, etc.) in preclinical safety studies.

#### 2.4.6. Comparative of Exosome Sources

Exosomes can be derived from a wide variety of cell types, and the choice of donor source profoundly influences their composition, bioactivity, and translational potential (Table 2). Among mammalian sources, mesenchymal stem cell (MSC)-derived exosomes are the most widely investigated due to their low immunogenicity, regenerative and anti-inflammatory properties, and scalable culture systems [28]. Immune cell-derived exosomes, particularly from dendritic cells and T cells, are valued for their antigen-presenting capacity and ability to stimulate or modulate immune responses, forming the basis for several cancer vaccine platforms [29]. In contrast, tumor-derived exosomes exhibit natural tropism toward tumor tissues and high intrinsic loading of oncogenic and signaling molecules; while useful as delivery models or biomarkers, their clinical translation is limited by potential immunosuppressive and pro-tumorigenic effects [30]. Epithelial and endothelial cell-derived exosomes are advantageous for vascular and barrier-related delivery, providing high yields and reproducible secretion [31].

Recently, non-mammalian sources such as plant-derived exosome-like nanovesicles and bacterial outer membrane vesicles (OMVs) have emerged as promising alternatives. Plant vesicles, obtained from sources like ginger, grapefruit, or broccoli, offer a safe, biocompatible, and potentially scalable platform for oral drug delivery [32]. Conversely, bacterial OMVs, while immunogenic, can be engineered to serve as vaccine carriers or adjuvants [33]. Collectively, the diversity of exosome sources provides a versatile toolkit for biomedical applications, but also underscores the need for rigorous source selection, purification, and functional validation to ensure safety and reproducibility.

## 3. Exosome Isolation and Characterization Techniques

Accurate and reproducible isolation and characterization of exosomes are critical for both fundamental research into intercellular communication and the development of exosome-based drug delivery systems. Exosomes, typically 30 nm–150 nm in diameter, exist in complex biological matrices co-present with other EV types, soluble proteins, lipoproteins, and nucleic acids making extraction nontrivial. Among standard approaches, ultracentrifugation (UC), whether as differential spin or density-gradient methods, remains widely employed. In differential UC, sequential low-speed centrifugations remove cells and debris, while high-speed spins at >100,000× *g* pellet small EVs. Despite its landmark status, differential UC often yields moderate purity, with co-sedimentation of protein aggregates and lipoproteins, and can induce structural disruption via shear forces. Density-gradient UC, for example, using sucrose or iodixanol cushions offers higher purity by separating vesicles by buoyant density, yet at the cost of lower yield, extended ultracentrifugation times, and laborious layering steps. Both formats require specialist instrumentation, rigorous protocol standardization, and can suffer from limited reproducibility across labs [34].

To overcome these challenges, size-exclusion chromatography (SEC) has gained traction as a gentle and reproducible alternative. In SEC, biofluids or concentrated samples are passed through columns packed with porous beads: larger particles such as exosomes elute in early fractions, while smaller proteins or aggregates are retained. Because SEC does not subject vesicles to high shear or osmotic pressure, it preserves structural and functional integrity. SEC yields higher purity than UC and maintains exosome bioactivity, while being both scalable and cost-effective no ultracentrifuge required making it attractive for clinical-grade exosome manufacturing (particularly when combined with upstream pre-concentration via ultrafiltration or tangential flow filtration) [35].

Ultrafiltration (UF), which employs membrane filters with tuned pore sizes, serves primarily as a rapid means to concentrate large fluid volumes and remove debris. With membranes layered by molecular weight cutoff or pore size (~0.1 µm–0.2 µm), UF can reduce sample volumes efficiently. However, unmodified UF on its own does not separate contaminants from EVs effectively and may incur membrane clogging or shear-induced deformation of vesicles. As a result, UF is frequently used as a preparative step prior to SEC or other purification techniques. Notably, tangential flow filtration (TFF) in which flow tangentially sweeps contaminants off the membrane surface has emerged as superior to dead-end UF in minimizing fouling and improving recovery, especially in scalable workflows meant for therapeutic exosome production [34].

Beyond physical separation, immunoaffinity-based isolation provides highly specific enrichment of exosome subtypes by targeting surface markers such as CD9, CD63, and CD81. Using antibody- or aptamer-functionalized magnetic beads or chromatography surfaces, this method can isolate defined exosome populations with exceptional purity ideal for biomarker discovery or functional studies. However, its output is typically low-yield and biased toward marker-positive subsets; costs per capture are high; and scaling to produce therapeutic quantities is limited. Thus, immunoaffinity is best suited for diagnostic or analytical applications rather than bulk drug delivery formulations [34].

Microfluidics-based platforms often termed lab-on-chip devices have innovated exosome isolation via size-based filtration, affinity capture, acoustic or electric field manipulation (e.g., dielectrophoresis, acoustofluidics), and integrated detection. These devices can simultaneously isolate and characterize exosomes from minute sample volumes, often delivering high purity and rapid processing times (minutes rather than hours). Examples include herringbone micromixers coated with anti-CD63, nano-structured deterministic lateral displacement chips, and magneto-electrochemical sensors coupled with immunoaffinity capture. These systems offer promise for point-of-care diagnostics and small-volume studies. However, their throughput is currently limited, scaling is immature, and device fabrication remains expensive and non-standardized, though as microfabrication matures, clinical-grade microfluidic platforms may become more viable for integrated EV workflows [36].

Increasingly, hybrid workflows combining multiple methods are favored. A common pipeline might begin with UF or TFF for concentration, followed by SEC for high purity, and optionally immunoaffinity capture for subtype-specific isolation. Such workflows can balance yield, purity, throughput, and functional preservation, and are consistent with the MISEV2018 guidelines, which recommend combining orthogonal separation modalities and testing for both positive (e.g., tetraspanins, TSG101, ALIX) and negative markers (e.g., nuclear or mitochondrial proteins) to assess contamination [37].

When comparing methods holistically, ultracentrifugation offers moderate yield and purity but risks vesicle damage and poor reproducibility; SEC, especially when paired with UF or TFF, delivers high purity and scalability; immunoaffinity capture offers specificity but limits throughput; and microfluidic platforms enable rapid, automated isolation but are not yet optimized for large-volume workflows. Therefore, context-specific decisions are necessary: preclinical work may tolerate hybrid UC/SEC workflows, while therapeutic exosome production demands strict reproducibility and purity (best met via SEC + TFF pipelines). For diagnostic biomarker discovery, immunoaffinity and microfluidics may be especially valuable. A comparison of different isolation methods is depicted in Table 3.

Physical loading methods such as electroporation and sonication can perturb exosomal membranes, with measurable consequences for how vesicles behave in vivo. Electroporation has repeatedly been shown to induce vesicle aggregation and alter basic surface properties, unless carefully buffered; these changes can impair the native ‘identity’ that guides biodistribution (e.g., increased size/aggregation and surface charge shifts that favor rapid uptake by the mononuclear phagocyte system) [38]. Beyond general colloidal effects, specific surface proteins are causally linked to organ tropism in vivo: for example, swapping exosomal integrins re-routes uptake to lung vs. liver and redirects metastatic seeding in mice, demonstrating that altered surface-marker presentation can profoundly change biodistribution and biological outcomes [39]. Protein corona remodeling can also retarget EVs in vivo—bound albumin was shown to steer EVs away from hepatic macrophages, further underscoring the sensitivity of biodistribution to surface chemistry [40].

Consistent with these mechanisms, sonication and related membrane-perturbing workflows have been reported to disrupt exosomal membranes and/or membrane proteins, which multiple reviews note can translate into altered in vivo distribution and efficacy if not controlled [41]. Mitigations exist: trehalose buffers reduce electroporation-induced aggregation, and optimized, low-stress electroporation protocols have achieved high drug loading and improved therapeutic responses without obvious loss of vesicle function, suggesting that careful parameterization can preserve integrity while gaining efficiency [38]. Altogether, available in vivo and mechanistic evidence supports that changes to exosomal surface markers or membrane properties—whether from cargo-loading or corona formation—can significantly affect biodistribution and therapeutic effect, and thus should be characterized (size, zeta potential, surface proteins) and, where possible, stabilized by formulation [42].

**Figure 3:**
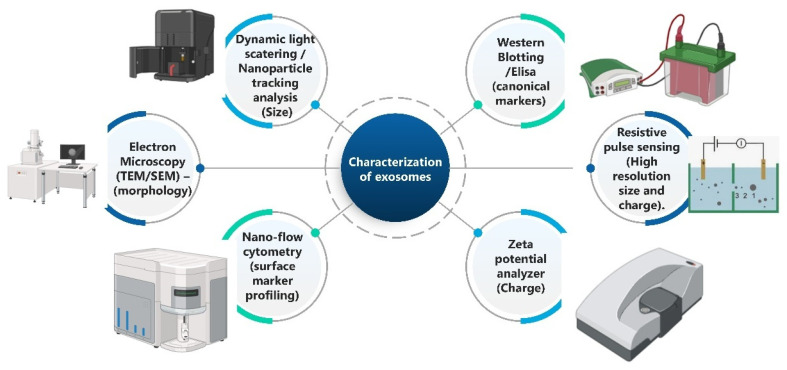
Characterization Techniques for Exosomes. Overview of commonly used methods for exosome characterization. The figure depicts biophysical techniques such as nanoparticle tracking analysis (NTA), dynamic light scattering (DLS), electron microscopy (TEM/SEM), and tunable resistive pulse sensing (TRPS), alongside biochemical approaches including Western blotting, ELISA, and flow cytometry for exosomal markers.

Following isolation, multi-modal characterization ensures exosome identity and quality (Figure 3). Nanoparticle tracking analysis (NTA) estimates size distribution and particle concentration by tracking Brownian motion under laser illumination. NTA provides rapid quantitative insight, though results can be sensitive to instrument settings and may over- or under-estimate size due to subdiffusive motion artifacts; correction tools like Finite Track Length Adjustment (FTLA) have been proposed to improve accuracy [43].

Transmission (TEM) and scanning electron microscopy (SEM) deliver nanoscale images of exosomes, confirming morphology (such as characteristic cup-shaped vesicles) and validating size. These techniques, however, are low throughput, time-intensive, and subject to sample preparation artifacts (e.g., dehydration, fixation). Thus TEM/SEM are best used to complement quantitative sizing data rather than serve as primary characterization tools.

Flow cytometry, especially specialized ‘nano-flow cytometry’ systems, enables surface marker profiling of exosomes at the single-vesicle level via fluorescent antibody labeling. Conventional cytometers struggle to resolve particles under ~200 nm and may suffer from “swarm detection” artifacts (multiple vesicles counted as one event). Nonetheless, where high-sensitivity instruments are available, flow cytometry can distinguish subpopulations by marker expression valuable in studying exosome heterogeneity across donor cell types or treatments [44].

Western blotting, ELISA, or mass spectrometry-based proteomics enable molecular-level validation. Western blotting is commonly applied to detect canonical markers such as CD9, CD63, CD81, ALIX, TSG101, and HSP70/HSP90 and to verify absence of contaminating markers (e.g., nuclear or mitochondrial proteins). Such marker profiling aligns with MISEV guidelines and is essential for confirming vesicle identity and purity, though it is not quantitative in terms of particle count [45].

Emerging techniques like tunable resistive pulse sensing (TRPS) measure size and zeta-potential via nanopore blockade, providing high-resolution size distribution and charge data. Super-resolution microscopy and Raman spectroscopy also enable single-vesicle-level molecular and structural profiling, though these remain primarily research tools at present [45].

Ultimately, standardizing exosome isolation and characterization workflows is a key hurdle for translation. The lack of uniform reference materials, interlaboratory variability, and absence of established GMP-compliant protocols all pose barriers. Integrating methods that preserve bioactivity, quantify yield, ensure marker verification and scalability is essential to advance exosome-based drug delivery from bench to bedside.

## 4. Strategies for Drug Loading into Exosomes

The potential of exosomes as therapeutic delivery vehicles has garnered immense interest due to their inherent biocompatibility, stability in circulation, low immunogenicity, and natural ability to traverse biological barriers such as the blood–brain barrier (BBB) [46]. A critical aspect of developing exosome-based drug delivery systems lies in efficiently loading therapeutic cargo ranging from small molecules and proteins to nucleic acids into exosomes. Broadly, drug loading strategies are categorized into endogenous (pre-secretory) and exogenous (post-secretory) methods. Each approach presents unique advantages and limitations in terms of efficiency, cargo stability, scalability, and translational applicability.

### 4.1. Endogenous Loading Strategies

Endogenous loading leverages the intrinsic cellular machinery to package therapeutic agents into exosomes during their biogenesis. This is typically achieved via either genetic engineering or passive incubation of donor cells with drugs.

Genetic Engineering of Donor Cells

One of the most precise and targeted approaches to loading exosomes is through the genetic engineering of parent cells, enabling selective incorporation of therapeutic molecules such as miRNAs, siRNAs, or therapeutic proteins. In this method, cells are transfected or transduced with plasmids encoding the desired therapeutic cargo, often fused with exosomal membrane proteins like CD63, Lamp2b, Alix, or TSG101, facilitating active sorting into exosomes [47].

For example, Alvarez-Erviti et al. demonstrated that exosomes derived from dendritic cells expressing Lamp2b fused with a neuron-specific RVG peptide could deliver siRNA specifically to the brain in mice, resulting in gene knockdown [48]. Similarly, Kojima et al. engineered exosomes to express synthetic RNA-binding domains, enabling programmable RNA loading through the EXOtic (EXOsomal transfer into cells) system [49].

One of the main advantages of this approach is its highly specific and programmable cargo loading, allowing for precise delivery of desired molecules. It also ensures stable incorporation during exosome formation, which enhances the reliability of the delivery system. Additionally, it enables the incorporation of hard-to-load cargo such as proteins or large RNAs, expanding the range of therapeutic applications. However, there are notable limitations. The process is time-consuming and technically complex, which may hinder widespread adoption. There is also potential safety concerns associated with the use of viral vectors, raising regulatory and ethical considerations. Furthermore, the method faces challenges with scalability, limiting its feasibility for clinical translation.

Passive Cargo Loading During Biogenesis

An alternative endogenous approach involves passive incubation of donor cells with small-molecule drugs or nucleotides. These agents are internalized by cells and subsequently encapsulated into exosomes via natural sorting mechanisms. For instance, Pascucci et al. loaded paclitaxel into mesenchymal stromal cells (MSCs), which then secreted drug-loaded exosomes with potent anti-tumor effects [50]. This approach is especially suitable for hydrophobic drugs that are easily internalized by cells and can integrate into exosomal membranes or lumens during exosome formation.

This method offers several advantages, including its simplicity and scalability, making it suitable for broader applications. It is non-invasive to the cellular machinery, minimizing potential disruption to cell function. Additionally, it avoids the use of complex vector systems, reducing technical challenges and safety concerns. However, it also presents some limitations. Loading efficiency is generally low and can be variable, which may affect consistency and effectiveness. There is also less control over cargo targeting or sorting, limiting precision in delivery. Moreover, there is a risk of drug degradation within the cells, which could compromise therapeutic outcomes.

### 4.2. Exogenous Loading Strategies

Exogenous or post-secretory loading involves manipulating isolated exosomes to encapsulate cargo after secretion. This strategy bypasses the complexities of cellular engineering and allows direct loading of purified exosomes.

Electroporation

Electroporation is one of the most widely used exogenous methods for loading nucleic acids into exosomes. By applying an electric field, transient pores are formed in the exosomal membrane, enabling cargo molecules particularly siRNAs or miRNAs to diffuse into the vesicle lumen. For instance, Wahlgren et al. successfully used electroporation to load siRNA into exosomes derived from human blood cells, demonstrating efficient delivery to recipient cells [51]. However, electroporation has been reported to cause exosome aggregation or siRNA precipitation, potentially compromising vesicle integrity and reducing cargo bioavailability [52].

This approach is particularly efficient for loading hydrophilic molecules such as RNA, making it valuable for nucleic acid-based therapies. It is also scalable and reproducible, supporting its potential for larger-scale applications. However, the method has several limitations. It may cause damage to exosomes or lead to their aggregation, which can affect their integrity and function. Loading efficiency can be inconsistent, posing challenges for standardization. Additionally, there is a risk of RNA degradation or precipitation during the process, which could reduce the effectiveness of the final therapeutic product.

Sonication

Sonication uses ultrasonic waves to disrupt the exosomal membrane, allowing passive diffusion of cargo into the vesicles. This mechanical method has been shown to significantly enhance drug loading efficiency, especially for small hydrophobic molecules. Haney et al. demonstrated that macrophage-derived exosomes sonicated with catalase had improved drug encapsulation and therapeutic efficacy in a Parkinson’s disease model [53].

This method offers high loading efficiency and is versatile, accommodating both hydrophilic and hydrophobic drugs, which broadens its therapeutic potential. However, it also presents important limitations. The process can cause structural damage to exosomes, potentially compromising their stability and function. It requires careful optimization to prevent degradation of the cargo, ensuring therapeutic efficacy. Additionally, the technique may alter surface markers or affect the biological activity of exosomes, which could impact targeting ability and safety profiles.

Incubation (Passive Loading)

Perhaps the simplest method, incubation involves mixing exosomes with the drug under physiological or mildly acidic conditions, allowing spontaneous diffusion of cargo into the exosome membrane or lumen. This method is particularly effective for small hydrophobic drugs like curcumin or paclitaxel [54]. For example, Sun et al. loaded curcumin into exosomes via passive incubation and reported enhanced anti-inflammatory activity in vitro [55].

This method is non-invasive and easy to implement, making it an attractive option for exosome loading. It preserves the integrity of exosomes, which is crucial for maintaining their natural structure and function. Additionally, it is scalable and compatible with Good Manufacturing Practice (GMP) standards, supporting its potential for clinical translation. However, the method has limitations, including low loading efficiency, particularly for hydrophilic molecules. The loading process is largely uncontrolled, which may affect consistency and reliability. Furthermore, it is not well-suited for macromolecules like RNA, limiting its applicability for certain therapeutic cargos.

Freeze–Thaw Cycles

This technique involves subjecting exosomes and cargo to cycles of freezing and thawing, which transiently disrupts the exosomal membrane, facilitating cargo encapsulation. This method is commonly used in conjunction with proteins and peptides [56]. This method is simple and does not require specialized equipment, making it accessible and easy to adopt in various settings. It is particularly suitable for loading peptides and proteins, offering a straightforward approach for incorporating biologically active molecules. However, it comes with several limitations. There is a risk of vesicle aggregation, which can affect the stability and function of the exosomes. Repeated loading cycles may lead to cargo leakage or degradation, reducing overall efficiency. Additionally, sensitive drugs are at risk of denaturation during the process, potentially compromising their therapeutic efficacy.

**Figure 4:**
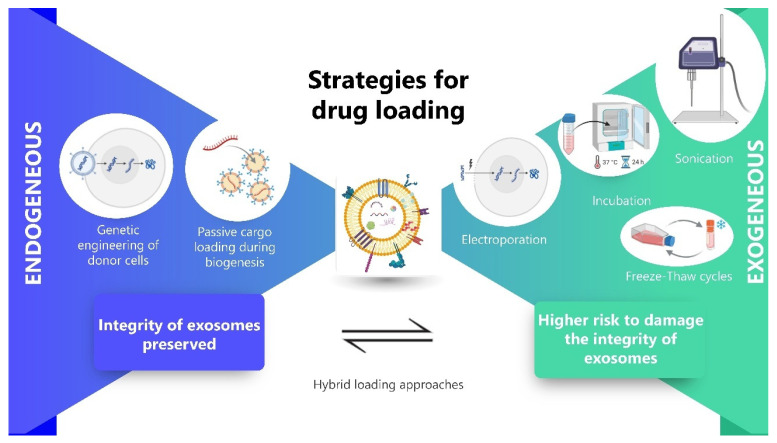
Endogenous vs. Exogenous Loading Strategies. Comparison of strategies for incorporating therapeutic cargo into exosomes. Endogenous loading involves modifying donor cells (e.g., transfection, drug incubation) so that exosomes are naturally secreted with desired cargo. Exogenous loading is performed post-isolation using techniques such as electroporation, sonication, extrusion, or chemical transfection.

Each loading technique presents a trade-off between efficiency, vesicle integrity, cargo stability, and translational scalability. A comprehensive comparison is summarized in Table 4:

Sonication and electroporation yield higher drug loading efficiency, but these methods may compromise the functional properties or surface markers of exosomes, affecting biodistribution and targeting. Conversely, endogenous methods, though labor-intensive, offer better control over cargo specificity and vesicle homogeneity. Furthermore, cargo type and intended therapeutic application often dictate the optimal loading strategy. For nucleic acid therapies targeting the central nervous system, electroporation combined with targeting ligand modification may be preferable. In contrast, hydrophobic small-molecule drugs can be effectively loaded via simple incubation methods without compromising exosomal integrity (Figure 4).

The choice of strategy is highly dependent on the nature of the therapeutic agent, desired targeting, and clinical application. A growing trend toward hybrid loading approaches combining endogenous and exogenous techniques is emerging to maximize efficiency and therapeutic efficacy. Moreover, advances in microfluidics, synthetic biology, and membrane engineering are expected to further refine loading technologies, enhancing the scalability, reproducibility, and clinical translatability of exosome-based drug delivery systems. Future research should also prioritize standardization of quantitative metrics for loading efficiency, stability assays, and in vivo functional validation to ensure consistent and safe clinical outcomes.

## 5. Targeting and Functionalization

Functionalization of exosomes through purposeful surface engineering and ligand design is central to unlocking their therapeutic potential. Tailoring exosome membranes to achieve cell-specific targeting, avoidance of off-target effects, and prolonged circulation is an active area of research and development. This section outlines the key strategies chemical, genetic, and hybrid for target-specific delivery to cancer cells, neurons, immune cells, and other therapeutic niches, while minimizing reticuloendothelial system (RES) clearance (Table 5).

### 5.1. Surface Engineering: Ligands, Peptides, and Antibodies

The exosomal membrane provides a versatile platform for surface display of targeting moieties, including peptides, antibodies, or aptamers. Such functionalization enhances specificity and uptake by desired cell types.

Chemical conjugation: Strategies include covalent attachment of ligands to surface proteins or lipids using click chemistry or EDC/NHS chemistry. Surface-engineered exosomes modified with antibodies (e.g., anti-HER2) or RGD peptides targeting αvβ3 integrins show significantly enhanced uptake by tumor cells both in vitro and in vivo, compared to unmodified controls [57].

Genetic engineering approaches: Producer cells can be transfected with plasmids encoding fusion proteins combining exosomal membrane proteins (such as Lamp2b or tetraspanins CD63/CD9) with targeting peptides (e.g., RVG for neuronal targeting or GE11 for EGFR-positive tumors). These engineered exosomes display ligands in orientation favorable for receptor binding and undergo uptake by target cells preferentially [58].

Hybrid nanoparticle approaches: Exosome–nanoparticle hybrids (e.g., gold, iron oxide, or silica nanoparticle cores) can be engineered with surface antibodies, peptides, or aptamers, augmenting both targeting ability and multimodal functions such as imaging, photothermal effects, or drug release. These hybrids offer combined benefits of exosome biocompatibility with nanoparticle tunability [59].

### 5.2. Cell-Specific Targeting: Cancer, Neurons, and Immune Cells

Target specificity is achieved by selecting ligands that bind receptors overexpressed on diseased or desired cell types:

- Cancer targeting

RGD peptides targeting αvβ3 and αvβ5 integrins are commonly used, as these receptors are overexpressed on tumor endothelium and aggressive tumor cells. RGD-functionalized exosomes achieve enhanced tumor uptake and deeper tissue penetration via receptor-mediated endocytosis [60]. Aptamer-functionalized exosomes targeting EGFR, nucleolin, or mucin-overexpressing cancer cells have shown high specificity and intracellular delivery of siRNA, as well as imaging agents in preclinical models [61]. Antibody-conjugated exosomes (e.g., anti-HER2, anti-CD44) further refine targeting and have demonstrated improved anti-tumor efficacy in xenograft models [62].

- Neuronal targeting

RVG peptide (rabies virus glycoprotein-derived) fused to Lamp2b enables selective delivery to neuronal and CNS cells via acetylcholine receptor binding. This strategy has been exploited to deliver siRNA across the blood–brain barrier in animal models of glioma and neurodegeneration [63]. Emerging efforts include designing exosomes with neuropilin-binding sequences or neural nanobody fusions to enhance CNS homing.

- Immune cell targeting

Exosomes surface-modified with ligands such as CD19, CD64, or immune checkpoint peptides enable targeting of B cells, T cells, or macrophages. These platforms are being explored both to deliver immunomodulatory cargo and to reprogram tumor-associated macrophages [59]. Macrophage targeting can also be facilitated by linking mannose or polysaccharides to exosome surfaces to leverage receptor-mediated endocytosis.

### 5.3. Avoidance of Off-Target Effects and RES Clearance

Effective targeting also means reducing off-target uptake particularly by phagocytes in the liver, spleen, or lung and prolonging systemic circulation:

CD47 “don’t-eat-me” strategy: CD47 protein expression on exosomes can inhibit macrophage-mediated clearance via SIRPα signaling. Producer cells overexpressing CD47, or post-isolation coating with recombinant CD47 or CD47-derived peptides, reduce clearance and increase circulation time in vivo [64].

PEGylation and stealth coatings: Similar to synthetic nanoparticles, polyethylene glycol (PEG) or zwitterionic polymers can be grafted onto exosome surfaces to reduce protein opsonization and uptake by Kupffer cells or splenic macrophages, though PEG may reduce cell uptake at the target site unless combined with targeting ligands [63].

Biomimetic coating strategies: Leveraging “self” markers such as CD47, CD55, or CD59, or piggybacking exosomes with red blood cell membrane fragments, can mimic autologous cells and evade immune surveillance while preserving targeting decorations [65].

Optimizing size and charge: Exosome size (~30–150 nm) and slight negative zeta-potential reduce rapid renal clearance and nonspecific uptake. Surface functionalization protocols must preserve these physical characteristics to avoid unintended biodistribution shifts.

### 5.4. Endosomal Escape and Intracellular Delivery

Efficient delivery of exosomal therapeutics relies not only on successful targeting and cellular uptake, but also on effective cargo release into the cytoplasm, where the therapeutic agents exert their function. One strategy involves the use of fusogenic peptides, such as GALA or H5WYG, displayed on the exosomal surface. These peptides undergo conformational changes under acidic endosomal conditions, disrupting the endosomal membrane and facilitating the escape of the cargo into the cytosol. Another approach employs pH-responsive linkers or lipid modifications, which respond to the lower pH within endosomes to trigger the controlled release of therapeutic payloads directly inside target cells. Additionally, exosomes may be combined with membrane-disrupting agents, such as mild electroporation or saponin treatments, during the cargo-loading process. These methods can enhance cytosolic delivery by temporarily permeabilizing vesicle or endosomal membranes, although they must be carefully optimized to avoid compromising exosome integrity or biological activity [62].

### 5.5. Emerging Trends and Combinations

The field of exosome research is rapidly advancing toward multivalent functionalization, enabling more precise and effective therapeutic strategies. One emerging approach involves the design of dual-targeting exosomes, which simultaneously display cancer-targeting peptides and immune-cell-homing ligands to enhance both tumor specificity and immune modulation within the tumor microenvironment. In parallel, multifunctional exosome–nanoparticle hybrids are being developed, incorporating imaging tags alongside targeting ligands to enable theranostic applications combining real-time imaging, targeted delivery, and controlled payload release in a single platform [66]. Additionally, synthetic exosome mimetics, outfitted with natural exosomal lipids and engineered targeting peptides, are being optimized for scalable manufacturing and clinical translation, offering a promising route to overcome production and regulatory hurdles.

**Table 5:** Summary of main targeting strategies used for exosome functionalization [57,58,60,62,66,67].

Strategy	Targeting Modality	Applications	Advantages and Challenges
Genetic engineering (e.g., Lamp2b fusion)	Peptides fused to exosomal proteins	Neuronal delivery, EGFR-positive cancer	High specificity, biologically uniform; requires transfection and safety validation
Chemical conjugation	Antibody, RGD, aptamer, PEG groups	Versatile targeting across cell types	Flexible, easily tunable; may alter exosome properties or uptake
Aptamer-functionalized exosomes	SELEX-identified oligonucleotide ligands	Cancer biomarkers, cell-specific delivery	High affinity and synthesis ease; stability and loading efficiency may vary
CD47/CD self-marker display	Immune evasion coatings	Prolonged circulation, reduced RES uptake	Reduces clearance; balancing targeting vs. stealth is critical
Hybrid exosome–nanoparticles	Embedded targeting ligands and payloads	Theranostics, multimodal therapy	High functional versatility; more complex manufacturing

## 6. Therapeutic Applications

Exosome-based delivery platforms hold transformative potential across a spectrum of clinical indications. Their intrinsic properties biocompatibility, low immunogenicity, and ability to cross biological barriers coupled with engineering versatility, enable precise delivery of small molecules, RNAs, proteins, and gene-editing complexes. This section reviews the emerging applications in cancer therapy, neurological disease, inflammatory and autoimmune disorders, gene therapy, mRNA delivery, and regenerative medicine (Table 6).

### 6.1. Cancer Therapy: siRNA, miRNA, Chemotherapeutics

Exosome-mediated delivery of anticancer agents has been studied extensively in preclinical models. Doxorubicin (DOX) was loaded into mesenchymal stem cell–derived exosomes, which when engineered with targeting ligands, achieved enhanced tumor localization, cytotoxicity, and reduced off-target toxicity in vivo. More recently, exosomes loaded with DOX via sonication from human adipose-derived MSCs (Exo-Dox) induced robust apoptosis and inhibited tumor migration in breast cancer co-cultures, outperforming free DOX in models including CAF co-cultures and xenograft mice, with significantly reduced tumor size and lower IC_50_ values [54,68].

In addition to chemotherapies, exosomes have emerged as effective carriers for siRNA and miRNA cargo targeting oncogenes or drug-resistance pathways. Exosomes bearing tumor-suppressive miRNAs showed robust inhibition of proliferation and metastasis in models of lung, liver, and pancreatic cancer, often overcoming drug resistance mechanisms and enhancing chemosensitivity [59]. Exosome-mediated RNA delivery provides targeted, low-toxicity modulation of tumor growth across multiple cancer types [69].

Hybrid exosome–nanoparticle constructs, such as iron oxide-conjugated DOX exosomes (magnetized Exo-Dox), enable theranostic approaches combining MRI imaging, magnetic targeting, and chemotherapy, improving tumor accumulation and treatment efficacy [70]. These platforms highlight the potential of combining exosomal delivery with external guidance or imaging modalities.

### 6.2. Exosomes as Bridges in Infectious Disease

Exosomes function as critical mediators of intercellular communication during pathogen infection. Exosomes serve as bidirectional regulatory agents, capable of both promoting infection and mounting defenses, effectively acting as “bridges” that deliver molecular cargo to influence host–pathogen interactions [71].

Pro-infection Roles of Pathogen-Derived or Pathogen-Modified Exosomes

Exosomes influence infection through three principal mechanisms:

1. Mediating further infection by transmitting pathogen-related molecules: Exosomes isolated from infected host cells may carry viral or bacterial proteins, pathogen-derived mRNA or miRNA, and even viral particles. These cargos can infect naïve cells or modulate host gene expressions to facilitate pathogen spread. For instance, exosomes released from cells infected with HIV, hepatitis viruses, or intracellular bacteria transport viral RNA or proteins that enhance infectivity in recipient cells [71].
2. Contributing to immune escape: Packaging of viral antigens within exosomes may lead to subversion of host antigen presentation pathways, avoiding recognition by cytotoxic T cells. Exosomal transfer of HIV Nef, HCV components, or EBV-associated factors can downregulate immune signaling or permit infected cells to evade immune detection [72].
3. Suppressing immunity by favoring immune cell apoptosis: Some pathogen-laden exosomes induce apoptosis in immune cells such as T cells or macrophages. For example, HCV-infected cell exosomes can trigger programmed cell death in plasmacytoid dendritic cells, thereby disrupting cytokine production and innate response [71].

Anti-infection Roles: Exosome-Mediated Host Defense

Conversely, exosomes also promote anti-infection activities through:

1. Direct inhibition of pathogen proliferation: Host-derived exosomes may carry antiviral factors—including APOBEC3G from CD8^+^ T cells, interferon-inducing RNAs, or suppressive proteins—that reduce viral replication or budding. In HIV infection, CD8^+^ T cell exosomes suppress HIV transcription via protein-dependent mechanisms, while APOBEC3G-containing exosomes inhibit virus spread in infected targets [73].
2. Activating innate and adaptive immune responses: Exosomes from infected cells frequently contain pathogen-associated molecular patterns (PAMPs) that stimulate innate receptors on macrophages, dendritic cells, and NK cells. For instance, Mycobacterium tuberculosis-infected macrophages release exosomes bearing bacterial components which activate TNF-α, IL-1β, and type I interferon responses. Similarly, exosomal viral RNAs from HCV-infected cells can induce IFN-α release from plasmacytoid dendritic cells [73].

On adaptive immunity, exosomes can deliver microbial antigens to antigen-presenting cells, facilitating cross-priming of CD4^+^ and CD8^+^ T cells. In models with T. gondii and M. tuberculosis, exosomes boost antigen-specific T cell responses and promote B cell activation and antibody production [73].

Specific Examples Across Pathogens

Viral Exosomes (HIV, HCV, HBV, SARS-CoV-2, EBV)

HIV: Exosomes from infected cells convey viral miRNAs and proteins (e.g., Nef) promoting infection and immune downregulation; conversely, CD8^+^ T cell-derived exosomes carrying APOBEC3G suppress HIV replication in target cells [72].

HCV: Exosomes containing HCV RNA activate plasmacytoid DCs to produce IFN-α. Meanwhile, HCV components delivered via exosomes can evade immune detection and support viral persistence [73].

HBV: Similar bidirectional roles—HBV-derived exosomes may inhibit immune recognition, yet host-exosomes with antiviral RNA/protein cargo participate in immune activation and viral control [72].

SARS-CoV-2/COVID-19: Emerging evidence shows exosomes ferrying viral RNA or proteins may modulate inflammatory pathways; meanwhile, exosomal cargo from immune cells or infected tissues may serve as biomarkers and modulate antiviral immunity [8,74].

EBV: Exosomes bearing EBV-encoded dUTPase trigger NF-κB signaling and cytokine release, enhancing antiviral inflammation; but some EBV-associated exosomes dampen antigen presentation to aid persistence [73].

Bacterial and Parasitic Infections

Mycobacterium tuberculosis: Exosomes from infected macrophages contain mycobacterial proteins and lipids that stimulate TNF-α and T cell responses; they also assist recruitment of antigen-specific CD4^+^ and CD8^+^ T cells, enhancing bacterial clearance [73].

Toxoplasma gondii: Infection-induced exosomes deliver parasite antigens to host immune cells, facilitating T cell priming and protective immunity [73].

Conceptualizing Exosomes as “Bridges”

Drawing these mechanisms together, exosomes clearly function as bridges between pathogens and host cells. They mediate the transfer of pathogen-derived signals to uninfected cells, facilitating either the spread of infection or the evasion of immune detection. Simultaneously, exosomes also transmit host immune signals that enhance both innate and adaptive immune responses in distant tissues. In this way, exosomes occupy a dynamic and central role in infection biology, serving as conduits for both pathogen and host-derived factors that collectively influence disease progression and immune outcomes.

Given this duality, exosomes present both promising opportunities and significant challenges in therapeutic and diagnostic contexts. As diagnostics, exosomal RNAs and proteins have the potential to serve as reliable biomarkers for infections such as HIV, HBV, and SARS-CoV-2, offering insights into disease stage and immune status. Therapeutically, exosomes are being explored as delivery vehicles for antiviral RNAs, immunostimulatory agents, and therapeutic proteins, due to their natural biocompatibility, low immunogenicity, and ability to target specific cell types. Additionally, exosomes carrying pathogen antigens may act as innovative, cell-free vaccine platforms capable of eliciting targeted T cell responses [75].

However, despite their potential, several critical knowledge gaps remain. One major challenge is the difficulty of isolating exosomes cleanly from virions or other extracellular vesicles, which hampers the ability to study their specific functions with precision. Furthermore, more robust in vivo evidence is needed to validate whether exosome-delivered RNAs exert functional effects in recipient mammalian cells. Another key question lies in understanding the targeting specificity of exosomes—why certain exosomes preferentially fuse with specific immune cells—and how the sorting of molecular cargo within infected cells is regulated. Finally, current research has largely focused on viral and bacterial pathogens, leaving a substantial gap in our understanding of exosome roles in fungal, parasitic, and emerging infectious diseases, which represents a crucial frontier for future investigation [73].

### 6.3. Neurological Diseases: BBB Traversal and CNS Delivery

One of the most promising therapeutic frontiers for exosome-based systems lies in treating neurological disorders, given their unparalleled ability to cross the blood–brain barrier (BBB) and deliver therapeutic payloads into the central nervous system (CNS) with precision. Traditional therapies struggle with CNS delivery due to the restrictive nature of the BBB, yet engineered exosomes through natural tropism, surface functionalization, and optimized administration routes offer a powerful solution for diseases like Alzheimer’s, Parkinson’s, stroke, glioblastoma, and rare neurogenetic disorders.

Crossing the Blood–Brain Barrier

The BBB is formed by brain microvascular endothelial cells connected by tight junctions, together with astrocyte endfeet, pericytes, and neuronal support structures. It restricts passage of most large molecules and nearly all macromolecules (>400 Da) from the blood into brain tissue [76]. Exosomes, however, can access the brain via receptor-mediated transcytosis or through natural trafficking mechanisms. A defining study by Alvarez-Erviti et al. demonstrated that exosomes engineered with the RVG peptide fused to the exosomal membrane protein Lamp2b successfully delivered siRNA into neurons, microglia, and oligodendrocytes after systemic administration, achieving gene silencing in the mouse brain with minimal off-target effects [48]. This approach has since been extended across preclinical models to deliver peptides, miRNA, chemotherapeutics, and other bioactive molecules.

Routes of Administration

Systemic Intravenous (IV) Administration: Engineered exosomes delivered intravenously have been shown to cross the BBB and accumulate in brain tissue. In models of traumatic brain injury (TBI), stroke, or neurodegeneration, systemic delivery of MSC-derived exosomes led to reduced neuroinflammation, enhanced neurovascular remodeling, and improved functional outcomes [77]. However, unmodified exosomes may predominantly accumulate in the liver and spleen unless specifically targeted.

Intranasal (IN) Administration: This non-invasive route bypasses the BBB via olfactory and trigeminal neural pathways. In several animal studies, exosomes (e.g., curcumin-loaded, miRNA-containing) administered intranasally rapidly reached the olfactory bulb and widespread brain regions, attenuating inflammation and promoting remyelination or neuroprotection in models of Parkinson’s and multiple sclerosis [78]. This route offers significant translational promise for chronic neurological therapies.

Local or Direct Brain Delivery: While invasive, direct intracerebral or convection-enhanced delivery (CED) allows localized administration of exosomal payloads, particularly in brain tumors such as glioblastoma. Such methods have shown efficacy with bypassed systemic clearance, though clinical applicability remains limited by surgical risks [79].

Engineering for CNS Targeting

Advancements in exosome engineering have significantly enhanced the efficiency of brain-targeted delivery. One key strategy involves the use of brain-homing peptides such as rabies virus glycoprotein (RVG), which, when fused to exosomal surface proteins like Lamp2b, facilitate receptor-mediated transcytosis across the blood–brain barrier (BBB). Similarly, ligands targeting transferrin receptors (TfR), which are highly expressed on brain endothelial cells, have been employed to improve exosome trafficking into the central nervous system. Another promising approach utilizes integrin-binding peptides such as RGD, which, when displayed on the exosomal membrane, increase targeting efficiency to inflamed or ischemic brain regions by binding to integrins that are upregulated during injury. These bioengineering strategies collectively enhance receptor-mediated uptake, promote more favorable biodistribution profiles, and reduce off-target accumulation in peripheral organs [80].

Therapeutic Applications in CNS Disorders

Exosome platforms have advanced in early preclinical stages across multiple neurological diseases:

Alzheimer’s Disease (AD): EVs loaded with siRNA targeting pathological proteins (e.g., BACE1 or tau) delivered via RVG-functionalized exosomes reduced amyloid burden, attenuated neuroinflammation, and improved cognitive performance in transgenic mouse models [81].

Parkinson’s Disease (PD): Intranasally administered catalase-containing exosomes overcame oxidative stress-induced neurotoxicity in MPTP mouse models and attenuated motor deficits. MSC-derived exosomes have also been used to deliver miRNAs that modulate alpha-synuclein aggregation and neuronal survival [78].

Glioblastoma and CNS Cancers: Exosome-mediated delivery of siRNA or chemotherapeutic agents (e.g., DOX, temozolomide) into glioblastoma-bearing mice resulted in tumor growth inhibition with reduced systemic toxicity. Strategies pairing targeting ligands with exosome carriers facilitate precise tumor homing [79].

Stroke and Traumatic Brain Injury: Systemic delivery of neurotrophic factor-loaded EVs (e.g., BDNF, NGF, miR-124) via intravenous or intranasal routes significantly improved neurological recovery and reduced infarct volume in rodent stroke models [77].

Neurogenetic Disorders: Exosome-based strategies delivering CRISPR/Cas9, mRNA, or RNAi have been tested in mouse models of Huntington’s disease and Amyotrophic Lateral Sclerosis, enabling gene modulation within the brain and prolonging survival in early studies [82].

### 6.4. Inflammatory and Autoimmune Diseases–Immune Modulation

Exosomes—especially those derived from mesenchymal stem cells (MSCs) or perinatal sources—have demonstrated remarkable immunomodulatory capacity, making them attractive candidates for treating inflammatory and autoimmune diseases. These vesicles carry enriched cargo such as miRNAs (e.g., miR-16-5p, miR-146a, miR-223-3p), proteins, and cytokines that shift immune responses from pro-inflammatory (M1) to anti-inflammatory (M2) states. For example, MSC-derived exosomes loaded with miR-16-5p suppress LPS/IFN-γ-induced polarization of macrophages into the inflammatory M1 phenotype, instead promoting M2 differentiation, which aids tissue regeneration in lung injury models [83]. Another review highlights how MSC-exosomes modulate signaling pathways such as JAK/STAT via exosomal miR-146a and miR-125a-3p, enhancing regulatory T-cell expansion, attenuating inflammation, and restoring immune homeostasis in systemic lupus erythematosus (SLE) and ulcerative colitis models [84].

In experimental models of inflammatory bowel disease (IBD), MSC exosomes have been shown to dampen dendritic cell activation, suppress pro-inflammatory cytokine release, and stimulate intestinal regeneration by activating receptors such as FXR and modulating NF-κB–mediated pathways [85]. In rheumatoid arthritis, exosomal treatments loaded with key miRNAs like miR-146a or miR-223-3p have rebalanced T-helper subsets, decreased Th17-driven inflammation, and promoted cartilage protection as reviewed in recent studies [84].

Critically, exosomes can also be engineered for targeted immune cell delivery. Exosomes decorated with surface ligands or loaded with specific anti-inflammatory miRNAs can home to macrophages or dendritic cells, reprogramming them into M2 phenotypes and dampening autoimmune flare-ups. This engineered modulation avoids the risks associated with cell therapy, such as potential tumorigenicity or immunoreactivity, while offering targeted suppression of pathogenic immune pathways [84].

Overall, preclinical evidence shows that exosome therapies can reduce inflammatory cytokines (e.g., TNF-α, IL-6, IL-1β), promote tissue repair, and shift immune dynamics toward long-lasting tolerance. Their multifunctional cargo and flexible engineering make them powerful tools against conditions such as IBD, lung injury, SLE, arthritis, and beyond [86].

### 6.5. Gene Therapy: Delivery of CRISPR-Cas9 Systems

Exosomes are emerging as a promising class of non-viral vectors for delivering gene-editing tools such as CRISPR-Cas9, offering distinct advantages in terms of safety, specificity, and delivery efficiency. Unlike viral systems, exosomes present lower immunogenicity, avoid insertional mutagenesis, and enable transient expression of gene-editing machinery—factors that reduce the risk of off-target effects and immune complications. In a pivotal study, Wan et al. (2022) demonstrated the therapeutic efficacy of exosome-delivered CRISPR-Cas9 ribonucleoprotein complexes (RNPs) in preclinical models of acute liver injury, fibrosis, and hepatocellular carcinoma (HCC) [87]. Their approach achieved efficient gene editing, restored liver function, and inhibited tumor progression, with fewer off-target mutations than traditional viral delivery methods.

Complementary to this, Gee et al. (2020) developed the NanoMEDIC system—a novel extracellular vesicle (EV)-based CRISPR delivery platform that uses a dual-homing mechanism to achieve transient and targeted genome editing [88]. By restricting Cas9 expression temporally, NanoMEDIC reduces long-term off-target risks and immunogenicity, presenting a safer alternative for in vivo applications. In oncology models, tumor-derived exosomes have been exploited for targeted delivery of CRISPR-Cas9 cargo, leveraging the intrinsic tropism of these vesicles toward their tissue of origin [89].

Recent reviews have thoroughly assessed the potential of exosome-based gene editing systems, highlighting key advantages such as high biocompatibility, targeted tissue delivery, and reduced innate immune activation [89]. However, they also emphasize several ongoing challenges, including difficulties in exosome loading efficiency, limited cargo capacity, batch-to-batch variability in production, and the need for scalable, GMP-compliant manufacturing protocols. Strategies such as electroporation, transfection of parent cells, or the use of membrane fusion peptides are being investigated to enhance CRISPR component encapsulation within exosomes while preserving functional activity. Moreover, synthetic exosome mimetics and hybrid nanovesicles are being developed to overcome natural exosomes’ production and stability limitations.

Together, these findings position exosome-mediated CRISPR-Cas9 delivery as a next-generation tool for in vivo gene therapy, with strong therapeutic potential across a range of diseases, including cancer, metabolic disorders, and genetic liver diseases. Nevertheless, translating these findings into clinical applications will require continued optimization of delivery specificity, dose control, and regulatory safety standards.

### 6.6. Regenerative Medicine: Tissue Repair and Cosmeceuticals

In regenerative medicine, exosomes—particularly those derived from mesenchymal stem cells (MSCs), neural stem cells (NSCs), and perinatal tissues such as amniotic fluid and umbilical cord—have gained recognition as key mediators of tissue repair and cellular restoration. These extracellular vesicles carry a diverse cargo of bioactive molecules, including mRNAs, microRNAs (miRNAs), cytokines, growth factors (e.g., VEGF, FGF, IGF), and matrix remodeling enzymes, which collectively modulate cell behavior, enhance paracrine signaling, and facilitate regeneration across a wide range of tissues.

Preclinical studies in myocardial infarction models have shown that intravenous or intramyocardial administration of MSC-derived exosomes enhances angiogenesis, reduces cardiomyocyte apoptosis, and improves left ventricular function without the tumorigenic or immunological risks associated with the cell [90]. Similarly, in diabetic wound healing, topical or injectable application of exosomes derived from adipose- or bone marrow-derived MSCs accelerates re-epithelialization, collagen deposition, and capillary network formation by activating PI3K/AKT and Wnt/β-catenin pathways [91]. In osteoarthritis, intra-articular injection of exosomes modulates macrophage polarization and reduces inflammatory cytokines, while simultaneously promoting chondrocyte proliferation and cartilage matrix synthesis [92].

The use of exosomes in spinal cord injury and traumatic brain injury models has also demonstrated promising neuroprotective effects. Neural stem cell-derived exosomes promote neurogenesis, axonal regeneration, and synaptic remodeling, likely mediated through miR-21, miR-124, and BDNF signaling pathways [93].

Cosmeceutical Applications and Skin Regeneration

Beyond traditional regenerative medicine, exosomes are making significant inroads into cosmeceuticals, particularly in skin rejuvenation, anti-aging, and scar repair. Skin aging is characterized by a decline in dermal fibroblast activity, collagen production, and epidermal barrier function, often driven by oxidative stress, UV exposure, and chronic inflammation. Exosomes derived from human dermal stem cells, adipose-derived MSCs, and placental tissues have been shown to reverse these effects by delivering key regenerative molecules such as TGF-β, EGF, miR-29, and collagen-stabilizing proteins, which stimulate fibroblast proliferation, elastin synthesis, and extracellular matrix (ECM) remodeling [94].

In vitro studies reveal that exosome treatment increases type I collagen synthesis and decreases matrix metalloproteinases (MMP-1, MMP-9)—enzymes involved in collagen degradation—thereby promoting dermal thickness and firmness. Additionally, exosomes reduce UVB-induced apoptosis in keratinocytes and melanocytes, suggesting their potential in photoprotection and pigment regulation [95,96].

In clinical cosmeceutical applications, topical formulations or microneedle-assisted delivery of exosome-based serums are being explored for skin brightening, wrinkle reduction, and wound healing. A randomized controlled trial using exosomes derived from human umbilical cord MSCs showed significant improvements in periorbital wrinkles, skin hydration, and elasticity after just four weeks of treatment, with no reported adverse events. The mechanism is thought to involve the suppression of inflammatory cytokines such as TNF-α and IL-6, alongside the activation of fibroblasts and epidermal stem cells [96].

Additionally, exosomes are being integrated into post-procedural recovery for dermatologic interventions such as laser resurfacing, chemical peels, and microneedling, where they help mitigate inflammation and expedite epithelial regeneration. Commercial cosmeceutical products, such as those containing EXO-SKIN or ASCE+ (Advanced Stem Cell Exosomes), are now being marketed with claims of clinical efficacy, though rigorous peer-reviewed validation remains an ongoing need.

## 7. Advantages of Exosome-Based Systems

Biocompatibility and Low Immunogenicity

One of the key strengths of exosomes as drug delivery vehicles lies in their endogenous origin they are naturally secreted by cells and composed of native lipids, proteins, and nucleic acids, which make them inherently biocompatible and minimally immunogenic (Table 7). Unlike synthetic nanoparticles or viral vectors, exosomes avoid provoking adverse immune reactions, even upon repeat administration [97]. This low immunogenicity enables safer and more prolonged systemic exposure, reducing the risk of clearance and anti-carrier immune responses. Indeed, exosome-based delivery systems have demonstrated significantly reduced toxicity and immune activation, offering a safer profile particularly important for chronic or repeated treatments in fields like oncology and neurology [98].

Intrinsic Targeting and Tissue Homing

Exosomes possess natural targeting capabilities derived from surface proteins and lipids that reflect their cell of origin. These molecular signatures—such as tetraspanins, integrins, and other adhesion molecules—can mediate specific interactions with recipient cells or tissues, enabling homing without the need for external modifications [99]. Such intrinsic targeting supports both passive and active delivery. Passive targeting occurs through biodistribution and physiological tropism, particularly in tumors or inflamed tissues. Active targeting can arise from exosomes sourced from specific cells; for example, exosomes derived from brain-homing cells may preferentially deliver cargo to the central nervous system. Exosome delivery can occur across barriers such as the blood–brain barrier (BBB), which is notoriously difficult for other carriers to penetrate. This natural tropism reduces off-target effects and enhances delivery precision, making exosomes attractive for therapeutic areas like neurodegeneration, oncology, and organ-specific regenerative medicine [99].

Enhanced Cargo Protection and Circulation Time

A vital advantage of exosome carriers is their lipid bilayer encapsulation, which shields sensitive therapeutic cargo such as RNA, protein, or small molecules from degradation by enzymes, pH changes, or serum nucleases in circulation [98]. Compared to free cargo or cargo encapsulated within synthetic nanoparticles, exosome-loaded molecules retain structural integrity and bioactivity for longer durations. This encapsulation also helps promote endosomal escape and efficient functional delivery to the cytoplasm of recipient cells [67]. Furthermore, exosomes exhibit longer circulation half-lives relative to many synthetic nanocarriers. This extended presence in the bloodstream enhances the probability of reaching target tissues, increases dosing flexibility, and reduces the frequency of administration required for therapeutic efficacy [65].

Versatility Across Cargo Types

Exosomes can carry a broad spectrum of therapeutic agents, including nucleic acids (mRNA, miRNA, siRNA), proteins, peptides, and small molecules. This versatility allows exosome platforms to accommodate complex biologics and gene therapies that often suffer from poor delivery efficiency in traditional systems [100]. For example, exosome carriers are being engineered for delivery of CRISPR-Cas RNPs, enabling genome editing in neurological disease models, something conventional vectors struggle to achieve with precision and reduced immunogenicity [67].

Ability to Cross Physiological Barriers

One of the most notable features of exosomes is their ability to penetrate physiological barriers, particularly the BBB. This property allows delivery of therapeutic agents to the brain via systemic administration, unlocking opportunities in neurodegenerative disorders such as Alzheimer’s, Huntington’s, and Parkinson’s diseases. Additionally, exosomes have demonstrated efficient tissue permeation in models of tumor targeting, organ regeneration, and cardiovascular repair. This capability arises from their small size (30 nm–150 nm), natural trafficking routes, and interactions with target cell receptors [101].

Scalability and Engineering Potential

Advances in bioengineering and surface engineering have enabled the development of engineered exosomes with tuned tropism, modified surface ligands, pH-sensitive release motifs, or magnetic guidance yet still preserving the biocompatible and low-immunogenic core [67].

Moreover, several groups are improving manufacturing processes to support scalable, reproducible GMP-grade production of exosomes with controlled cargo loading and batch consistency. Combined with advances in microfluidic isolation, ultrafiltration, and size-exclusion techniques, these manufacturing innovations strengthen the industrial viability of exosome platforms [102].

## 8. Limitations and Challenges

Although exosome-based drug delivery systems offer promising advantages, their translation into clinical use remains impeded by significant technical, production, and regulatory hurdles. The key challenges include low isolation yield and purity, lack of standardized production protocols, limited loading efficiency and reproducibility, regulatory and scalability issues, and the potential for immunological effects depending on the exosome source (Table 8).

Isolation Yield and Purity

A fundamental barrier facing exosome research is the inherently low yield and inconsistent purity of exosome preparations. Typical isolation from cell culture media yields less than 1 µg of exosome protein per mL of conditioned medium, whereas therapeutic dosing in preclinical animal studies often requires tens to hundreds of micrograms per subject [103]. Common isolation techniques—such as ultracentrifugation, ultrafiltration, size-exclusion chromatography, or polymer precipitation—frequently co-isolate contaminants including protein aggregates, lipoproteins, and cellular debris, compromising exosome purity and downstream functionality [103]. These limitations impose high input requirements, increase cost, and limit scalability for therapeutic applications.

Lack of Standardized Protocols

The fragmentation of methodologies across exosome studies undermines comparability and reproducibility. There is no universally accepted protocol for critical steps such as cell source selection, culture conditions, isolation, quantification, or quality control—resulting in high variability in exosome composition and bioactivity between labs and batches [104]. Such heterogeneity impedes establishment of reliable potency assays and comparability across preclinical and clinical studies. Recognizing this, professional bodies like the International Society for Extracellular Vesicles (ISEV) and others have called for standardized reporting of key experimental parameters and adherence to minimal information guidelines [105].

Limited Loading Efficiency and Reproducibility

Efficient and consistent cargo loading remains a major technical challenge. Passive techniques like simple incubation tend to result in low encapsulation rates (often <10%), highly influenced by the physicochemical properties of the cargo and limited by non-specific associations rather than true luminal loading [65]. More active methods like electroporation, sonication, detergent permeabilization, and freeze/thaw can improve cargo load but introduce issues such as RNA degradation, vesicle aggregation, vesicle damage, or inconsistent loading across batches [105]. For example, electroporation may result in RNA aggregation and particle aggregation, reducing actual loading below 0.05% in some cases [104]. Reproducibility remains low in part because standardized metrics and experimental details such as EV-to-cargo ratios, temperature/time profiles, or post-loading purification are often incompletely reported [105].

Regulatory and Scalability Challenges

Clinical translation requires reliable large-scale GMP production systems with rigorous control of quality, potency, and safety. However, scaling current laboratory-based workflows to manufacturing scale is challenging due to low yield, heterogeneous product, and the absence of robust potency or batch-release assays [106]. Regulatory bodies—such as the FDA, EMA, and national agencies—have yet to establish specific guidelines tailored for exosome-based products, meaning developers must align with broader cell therapy and biologics frameworks. This creates ambiguity regarding classification (as biologics, ATMPs, or drug delivery systems), as well as for required testing—including characterization of identity, purity, potency, biodistribution, pharmacokinetics, dose-finding, immunogenicity, and tumorigenicity [107]. Without standardized safety/efficacy endpoints, translation is slowed by inconsistent regulatory expectations across jurisdiction.

Potential for Immunological Effects Depending on Source

While exosomes are often lauded for low immunogenicity, immune responses may still arise depending on the cell source or purification quality. Serum-derived or MSC-derived exosomes can carry immunomodulatory molecules (MHC proteins, cytokines, small RNAs) that may trigger unintended immune activation or tolerance. Moreover, contaminating proteins or residual cell components can act as immunogens if not removed thoroughly [103]. Additionally, differences in biodistribution and clearance such as uptake by the reticuloendothelial system (RES) are influenced by membrane composition and zeta-potential, potentially triggering rapid clearance or inflammatory responses [65]. Without full profiling of exosome surface proteins and cargo, immune safety remains an open question.

Stability, Storage, and Shelf Life

Another understudied yet critical challenge is stability—many formulations of exosomes are stored at ultra-low temperatures (e.g., −80 °C), which is impractical for commercial or clinical deployment. Publication-level data on shelf-life, formulation stability, or stability under typical shipping and handling conditions is largely lacking. Such gaps pose hurdles for translation into off-the-shelf products and influence decisions around lyophilization, cryopreservation, or buffer formulation [65].

Barriers to Clinical Translation

Among these challenges, the most significant current barrier is manufacturing scale-up and standardization for clinical-grade production. Conventional isolation methods such as ultracentrifugation or polymer precipitation typically yield heterogeneous vesicle populations of limited purity, which undermines batch-to-batch reproducibility and complicates compliance with Good Manufacturing Practice (GMP) requirements. Without robust scalable technologies (e.g., bioreactor-based production, tangential-flow filtration, microfluidics), achieving consistent therapeutic doses is not feasible for late-phase clinical trials. Closely linked to this are regulatory acceptance hurdles, since agencies such as FDA and EMA require clear criteria for product identity, potency, and release testing—standards that are difficult to establish without reliable large-scale production platforms. In contrast, targeting specificity, although an important determinant of therapeutic efficacy, is advancing rapidly through surface engineering strategies, ligand display, and exosome–nanoparticle hybrids. Therefore, while all three remain relevant, the primary bottleneck for translation is manufacturing and regulatory readiness, with targeting specificity considered a more tractable technical challenge under active development [108].

Strategies to Overcome These Challenges

**Figure 5:**
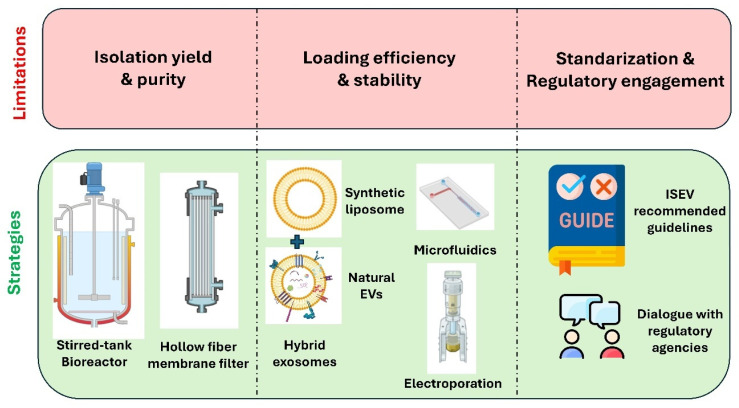
Limitations of Exosome Therapeutics and Strategies to Overcome Them. Schematic summary of the key limitations of exosome-based drug delivery—including low yield, heterogeneous populations, rapid clearance, and limited targeting specificity—and strategies under investigation to address these challenges. Solutions depicted include scalable bioreactor production, advanced purification methods, surface engineering for improved tropism, and hybrid exosome–nanoparticle systems.

To improve the clinical viability of exosome therapeutics, several strategies are emerging (Figure 5):

Enhanced production platforms: Utilization of bioreactors (e.g., stirred-tank, hollow-fiber, suspension systems) and optimized cell culture media enhances exosome yield while improving batch consistency [98].

Standardized protocols and reporting: Adoption of ISEV-recommended guidelines for minimal data reporting and methodology will help improve experimental rigor and cross-study comparability [105].

Hybrid and engineered exosomes: Membrane-hybrid approaches that combine synthetic liposomes and natural EVs may improve cargo loading and stability while preserving targeting capability [65].

Optimized active loading strategies: Refinements in electroporation parameters, microfluidic loading, or novel permeabilization tools may increase reproducibility while limiting cargo degradation or vesicle damage [105].

Regulatory engagement: Early and sustained dialogue with regulatory agencies to co-develop guidance on product classification, potency assays, and acceptable quality attributes will foster translational progress [107].

While exosome-based systems deliver compelling advantages in drug delivery, their translation is limited by multiple challenges including low and impure yields, inconsistent production protocols, weak cargo-loading reproducibility, unclear regulatory pathways, potential immunogenicity, and poor stability. Addressing these limitations through technological innovation, standardized practices, and regulatory alignment is essential. As the field matures, coordinated efforts across academia, industry, and regulatory stakeholders will be key to unlocking the full therapeutic potential of exosome-based therapeutics.

## 9. Market for Exosomes and Regulatory Challenges

A growing number of biotechnology companies are advancing exosome-based therapeutics and establishing scalable manufacturing platforms for clinical use (Table 9). Aegle Therapeutics (Miami, FL, USA) is a regenerative medicine company advancing extracellular vesicle (EV) therapies to address rare and debilitating dermatological diseases, notably Recessive Dystrophic Epidermolysis Bullosa (RDEB). Their lead candidate, AGLE-102, a composite of EVs including exosomes derived from bone marrow–mesenchymal stem cells (BM-MSCs), is currently in a Phase 1/2 clinical trial for RDEB. These EVs, isolated using Aegle’s proprietary harvesting technology, serve as key bioactive agents of MSCs, delivering proteins and nucleic acids (such as microRNAs and mRNAs) that influence tissue regeneration through paracrine signaling. This signaling enhances cell proliferation, migration, and survival, both locally and systemically. Notably, BM-MSC EVs are capable of transferring essential skin basement membrane proteins like collagen IV and VII, which are directly relevant to the pathology of RDEB. With MSCs known for their ability to differentiate into various mesodermal tissues, Aegle’s approach harnesses their regenerative potential through the targeted use of secreted EVs, offering a novel therapeutic avenue for cutaneous repair and broader systemic applications [109].

Capricor Therapeutics (San Diego, CA, USA) is advancing a multi-stage pipeline centered on allogeneic cardiosphere-derived cells (CDCs) and exosome-based therapies to address a wide range of serious diseases. Their lead candidate, deramiocel (CAP-1002), consists of CDCs stromal cells derived from healthy human heart tissue originally discovered by Capricor’s scientific founder, Dr. Eduardo Marbán, at Johns Hopkins University. CDCs have been extensively studied, featured in over 100 peer-reviewed publications, and administered in multiple clinical trials involving more than 200 patients. In the context of Duchenne muscular dystrophy (DMD), deramiocel exerts immunomodulatory, anti-inflammatory, pro-angiogenic, and anti-fibrotic effects, largely mediated through exosomes containing bioactive molecules such as microRNAs. These exosomal contents modulate immune responses, particularly by altering gene expression in macrophages, thereby reducing inflammation and promoting tissue regeneration. Capricor’s ongoing research also focuses on characterizing and expanding deramiocel’s potential, including its use in combination with other emerging DMD therapies to address the broader pathophysiology of this chronic inflammatory condition [110].

Evox Therapeutics (Oxford, UK) is pioneering the use of engineered exosomes to safely and effectively deliver genome-editing tools for the treatment of neurological and neurodegenerative diseases. Their approach combines the precision of CRISPR-Cas genome editing with the natural delivery capabilities and safety profile of exosomes, overcoming limitations associated with traditional CNS-targeting methods like viral vectors or lipid nanoparticles. Evox’s exosome-based platform enables targeted, transient delivery of Cas ribonucleoproteins, minimizing off-target effects, reducing immunogenicity, and allowing for safe re-dosing. These exosomes exhibit broad tissue tropism and effective neuronal penetration, making them ideal for CNS applications. Evox is developing therapies for diseases with well-defined genetic drivers, including Spinocerebellar Ataxia type 2 (SCA2) via ATXN2 targeting, and Huntington’s disease through MSH3 suppression, with potential relevance to other repeat expansion disorders. Their modular exosome platform supports diverse genome-editing technologies and is being expanded through strategic partnerships, aiming to unlock treatment options for a broad range of severe genetic conditions beyond the CNS [111].

Aruna Bio’s (Athens, GA, USA) lead therapeutic exosome, AB126, is uniquely engineered for central nervous system (CNS) specificity; its surface carries neural-derived receptors and proteins, enabling it to cross the blood–brain barrier and naturally home to brain regions such as the cerebellum and basal ganglia. In preclinical studies, AB126 exhibits innate therapeutic activity without additional payloads: it reduces neuroinflammation, protects neurons, and promotes neuroregeneration, including enhanced stem-cell proliferation and remyelination. Moreover, AB126 engages an anti-inflammatory mechanism via degradation of extracellular ATP into adenosine mediated by its intrinsic CD39/CD73 enzymatic markers supporting modulation of the immune cascade in both acute and chronic CNS disease models. The FDA has cleared its IND for a Phase 1b/2a clinical trial in acute ischemic stroke, making AB126 the first exosome therapy to enter human trials for a neurological indication; enrollment is planned in patients with poor prognosis following thrombectomy. Aruna also piloted AB126 in preclinical ALS (SOD1) mouse models, where it extended survival, reduced inflammation, and lowered neurofilament light chain biomarkers after weekly dosing. Complementing its therapeutic profile, Aruna has developed a scalable cGMP manufacturing platform with proprietary expertise in exosome purification, concentration, and batch consistency, enabling production of clinical-grade material and eventual scale-up through Phase 3 and potential commercialization [112].

EverZom (Paris, France), founded in 2019 by researchers from CNRS and Université Paris Cité, is a pioneering biotech developer of exosome-based regenerative therapies, recognized as a winner of the prestigious European EIC Accelerator Program. Its mission is to become a leader in therapeutically leveraging exosomes across diverse clinical contexts. The company has built a comprehensive, patent-protected innovation platform covering the entire exosome value chain from cell sourcing and exosome generation to cargo loading and formulation. This platform is optimized for high-yield, scalable and reproducible manufacturing, including mechanical stimulation methods that boost exosome output by orders of magnitude and facilitate clinical-grade production. EverZom’s lead product, EVerGel, targets digestive tissue healing, specifically Crohn’s-related perianal fistulas and post-surgical anastomotic healing. The therapeutic combines stem cell-derived exosomes with a thermosensitive hydrogel to enable controlled, localized release. Preclinical studies across multiple animal models have shown rapid healing, reduced inflammation, and minimized fibrosis. IND-enabling studies are expected by late 2024, with first-in-human dosing anticipated in early 2026. A second candidate, administered intravenously, is designed to promote organ regeneration, beginning with treatment of liver failure in chronic insufficiency settings such as cirrhosis. EverZom’s platform has already validated the manufacture of its first GMP-grade exosome batch at scale in a 10 L bioreactor, developed in partnership with the French Blood Establishment (EFS), achieving unprecedented yields and paving the way to clinical translation by early 2025 [113].

Aposcience AG (Vienna, Austria) is a leading innovator in regenerative medicine, developing therapies based on the secretome, a potent, cell-free mixture of bioactive molecules secreted by apoptotic peripheral blood mononuclear cells (PBMCs). Unlike exosome-only therapies, which isolate a specific extracellular vesicle subtype, secretome-based therapies encompass a broader range of biologically active components including proteins, lipids, cytokines, growth factors, and exosomes, offering multi-modal regenerative effects. This comprehensive approach is designed to restore tissue function rather than merely repair damage. Aposcience’s core platform uses PBMCs that are driven into apoptosis through controlled stress (e.g., irradiation), triggering the release of a highly active secretome. This secretome has shown superior efficacy in promoting wound healing, skin regeneration, and recovery from ischemic inflammatory conditions such as myocardial infarction, stroke, and spinal cord injury. Two formulations are under development: APO-1 (systemic) and APO-2 (topical), both produced from the secretome of healthy donor blood using a proprietary process. These formulations enhance healing by stimulating keratinocyte and fibroblast migration, improving angiogenesis, and increasing oxygen and nutrient delivery to damaged tissue. The company’s flagship clinical program, APO-1, is currently being evaluated in the MARSYAS II multinational, randomized, double-blind clinical trial for the treatment of acute myocardial infarction (MI). In preclinical models, APO-1 significantly reduced infarct size and preserved heart function, both with and without reperfusion, demonstrating its potential to prevent adverse cardiac remodeling in over 700,000 patients globally who undergo reperfusion therapy annually. If successful, APO-1 could address a market opportunity of € 1–2 billion per year [114].

Codiak BioSciences (Cmabridge, MA, USA) was a recognized leader in this space [115]. Codiak’s exoASO-C/EBPβ was a novel therapeutic that selectively targets the transcription factor C/EBPβ in immunosuppressive myeloid-derived suppressor cells (MDSCs), leading to immune modulation and potent systemic anti-tumor activity across multiple MDSC-rich, checkpoint-resistant tumor models. By leveraging exosome surface glycoproteins, the therapy enables precise uptake of antisense oligonucleotide (ASO) payloads by MDSCs and monocytes, facilitating effective delivery to distant, extra-hepatic tumor sites. This targeted delivery results in modulation of the tumor microenvironment and activation of T-cell-mediated anti-tumor responses, which are further enhanced when combined with anti-PD1 checkpoint inhibitors. Preclinical studies demonstrated robust monotherapy efficacy across several administration routes, underscoring the platform’s potential applicability in various cancers. Transcriptomic analyses using data from The Cancer Genome Atlas (TCGA) further identified C/EBPβ and MDSC-related gene signatures enriched across diverse tumor types, supporting broad clinical relevance. In parallel, exoASO-STAT6, another candidate from Codiak’s exoASO™ platform, targets STAT6 in tumor-associated macrophages (TAMs), particularly within hepatic tumors such as hepatocellular carcinoma (HCC). Systemic administration in preclinical models resulted in durable, dose-dependent liver retention of the ASO and sustained downregulation of STAT6 mRNA, along with modulation of downstream immune pathways. STAT6 and IL-4 receptor (IL4R) expression in human HCC TAMs, along with the identification of a STAT6-related transcriptional signature associated with poor prognosis in HCC and other cancers, supports the rationale for clinical development [116]. However, the financial risk of exosome-based companies is high. For example, Codiak Biosciences filed for bankruptcy in 2023.

In the manufacturing domain, several companies are building GMP-compliant infrastructure to support clinical and commercial needs. EXO Biologics (Lieja, Belgium), headquartered in Liège, Belgium, is a leader in developing therapeutics based on mesenchymal stromal cell-derived exosomes. Its proprietary ExoPulse™ platform supports GMP-compliant production, purification, and characterization of exosomes for clinical use [117]. The company’s subsidiary CDMO, ExoXpert, was launched to meet growing global demand for clinical-grade exosomes. ExoXpert operates a state-of-the-art GMP facility utilizing ExoPulse™, offering full-scale production capacity for both internal projects and external partners. Their lead therapeutic candidate, EXOB-001, is an MSC-derived exosome formulation currently in an EMA-approved Phase I/II clinical trial (EVENEW) for the prevention of bronchopulmonary dysplasia (BPD) in preterm infants. This program represents the first European Medicines Agency-authorized exosome-based therapy in clinical development [118]. Additional milestones include grant of Orphan Drug Designation and Rare Pediatric Disease Designation by both EMA and FDA for EXOB-001, and ExoXpert receiving GMP certification from the Belgian medicines agency, enabling the first specialized exosome CDMO in Europe and demonstrating capability to reliably produce GMP-grade exosomes, including loading of mRNA and DNA payloads with up to ~80% retention, paving the path for exosome-based gene delivery applications [119].

Kimera Labs (Miramar, FL, USA) is a biotechnology company specializing in exosome-based innovations, with a strong commercial focus on topical skin health and cosmetic applications. Their flagship product, Vive, represents the culmination of years of exosome research, formulated specifically to support skin vitality, hydration, and youthful appearance. Vive contains 2 trillion microvesicles per 5 mL vial, including billions of biologically active exosomes, derived from mesenchymal stem cells. These exosomes act as cellular messengers, promoting intercellular communication that may support skin texture, elasticity, and regeneration. The formula also combines amino acids (the building blocks of proteins), hyaluronic acid (for deep hydration), and trehalose (for cellular protection and moisture retention), making it a multifunctional skin therapy [120]. Unlike pharmaceutical exosome platforms still in clinical development, Vive is already commercially available and widely used as a topical cosmetic product, often in conjunction with aesthetic procedures such as microneedling, laser resurfacing, or RF therapy. This positions Kimera Labs at the intersection of regenerative science and luxury skincare, offering consumers access to non-invasive, cell-derived regenerative treatments that tap into the biological power of exosomes for visible skin rejuvenation.

ExoCoBio’s (Geumcheon-gu, Seoul) Derma Signal SRLV (marketed as ASCE+ SRLV) is a next-generation topical skin regeneration treatment that leverages the therapeutic power of exosomes, nano-sized extracellular vesicles involved in cellular communication and repair. Utilizing ExoCoBio’s proprietary ExoSCRT™ purification technology, the exosomes in Derma Signal SRLV are highly purified and bioactive, enabling them to modulate skin repair processes at the cellular level. Clinical and preclinical data suggest impressive regenerative effects, including up to a 690% increase in collagen production, a 300% boost in elastin, and a 75% reduction in melanin, contributing to visibly firmer, brighter, and more even-toned skin. The treatment is particularly effective for deep skin rejuvenation, accelerated healing after cosmetic procedures (such as laser, microneedling, or chemical peels), and hydration enhancement, while also offering benefits for sensitive or inflamed skin conditions such as rosacea or acne. It helps reduce pigmentation, improve skin texture, and minimize scarring by promoting keratinocyte and fibroblast activity. Administered typically via microneedling, the recommended protocol involves a minimum of three sessions spaced 3–4 weeks apart, targeting the face, neck, and décolleté. Already in commercial use in aesthetic clinics, Derma Signal SRLV exemplifies how exosome science is being effectively translated into high-performance cosmetic dermatology [121].

RION (Rochester, MN, USA), founded in 2017 through the Mayo Clinic Employee Entrepreneurial Program and headquartered in Rochester, MN, is a clinical-stage regenerative medicine company pioneering a novel exosome therapeutic platform. Its core innovation, Purified Exosome Product™ (PEP™), is a shelf-stable lyophilized powder derived from human platelets and discovered within the Mayo Clinic’s Van Cleve Cardiac Regenerative Medicine Program [122]. PEP™ exosomes are engineered to accelerate tissue repair by promoting cell proliferation, angiogenesis, and inflammation resolution while stabilizing cellular microenvironment integrity. Leveraging a proprietary biomanufacturing platform, RION has scaled PEP™ production to clinical and commercial standards, supported by over 30 peer-reviewed publications and multiple IND-enabling preclinical programs across wound healing, musculoskeletal, cardiovascular, pulmonary, and women’s health indications. Clinically, PEP™ is advancing through human trials: a Phase 2A study in Diabetic Foot Ulcers (DFUs) commenced dosing in May 2024, enrolling 59 patients and focusing on safety and healing efficacy versus standard care. A separate Phase 1b trial launched in early 2025 evaluates intra-articular injection of PEP™ for knee osteoarthritis, with enrollment initiated in March 2025 across multiple U.S. centers. RION’s platform exemplifies how platelet-derived exosomes can transform regenerative medicine through off-the-shelf, clinically scalable solutions that empower the body’s innate healing mechanisms [122].

In addition to product developers, contract manufacturing organizations like SCTbio (Praha, Czech Republic) and Lonza (Basilea, Switzerland) are providing essential GMP production and testing services to support exosome therapeutics at scale [123,124]. RoosterBio (Frederick, MD, USA), in collaboration with Thermo Fisher Scientific (Waltham, MA, USA), offers engineered MSC platforms and bioprocess tools to streamline exosome manufacturing workflows for clinical-grade applications [125].

Collectively, these companies are laying the foundation for the clinical and commercial translation of exosome-based therapies, addressing key challenges in scalability, reproducibility, and regulatory compliance while expanding therapeutic possibilities across oncology, regenerative medicine, infection and rare diseases.

**Table 9:** Leading companies in the manufacturing of exosomes.

Company	Focus Area	Lead Candidate	Modality	Development Stage	Ref
Aegle Therapeutics	Dermatology (RDEB)	AGLE-102	BM-MSC-derived EVs	Phase 1/2 Clinical Trial	[109]
Capricor Therapeutics	Muscular dystrophy, Inflammation	Deramiocel (CAP-1002)	Cardiosphere-Derived Cells (CDCs) and Exosomes	Clinical Trials (DMD, others)	[110]
Evox Therapeutics	Neurological diseases (e.g., SCA2, HD)	Genome-editing exosome platform	Engineered Exosomes + CRISPR-Cas RNPs	Preclinical/Strategic partnerships	[111]
Aruna Bio	CNS (e.g., Stroke, ALS)	AB126	Neural Exosomes with BBB penetration	Phase 1b/2a Clinical Trial	[112]
EverZom	Digestive healing, Organ regeneration	EVerGel	Stem cell-derived Exosomes + Hydrogel	Preclinical; IND-enabling by 2024	[113]
Aposcience AG	Cardiovascular, Skin regeneration	APO-1, APO-2	PBMC-derived Secretome (incl. Exosomes)	Phase 2 (MI), Preclinical	[114]
Codiak BioSciences	Oncology (Checkpoint-resistant tumors)	exoASO-C/EBPβ, exoASO-STAT6	Engineered Exosomes + ASOs	Preclinical (Bankruptcy in 2023)	[116]
EXO Biologics/ExoXpert	Neonatal lung disease (BPD)	EXOB 001	MSC-derived Exosomes	Phase 1/2 Clinical Trial (EMA-approved)	[119]
Kimera Labs	Cosmetics, Skin health	Vive	Topical MSC-derived Exosomes	Commercial (Cosmetic Use)	[120]
ExoCoBio	Aesthetic Dermatology	Derma Signal SRLV (ASCE+)	Topical Exosomes (ExoSCRT™ tech)	Commercial (Aesthetic Clinics)	[121]
RION	Regenerative medicine	PEP™	Platelet-derived Exosomes (Lyophilized)	Phase 2A (DFU), Phase 1b (OA)	[122]
RoosterBio/Thermo Fisher	GMP Manufacturing Tools	MSC + Bioprocess Platforms	Clinical-grade MSC-derived Exosomes	Manufacturing Support	[125]
SCTbio, Lonza	Contract Manufacturing	Not applicable	CDMO Services for Exosomes	GMP-compliant manufacturing	[123,124]

In addition to the well-established oncology and regenerative medicine programs already discussed, several emerging or exploratory clinical studies illustrate the widening therapeutic scope of exosome-based interventions. Trials such as NCT02310451 investigated the role of exosomes in melanoma pathogenesis, underscoring their diagnostic and mechanistic relevance in cancer biology. Other studies have explored regenerative and anti-inflammatory applications, including NCT04270006 (adipose-derived stem cell exosomes for periodontitis) and NCT06853522 (human umbilical cord MSC-derived exosomes for active ulcerative colitis). Novel neuromodulatory approaches have also been initiated, such as NCT04202783, assessing exosomes for craniofacial neuralgia, and a recent project combining focused ultrasound with exosome delivery for depression, anxiety, and dementias. Cosmetic and dermatologic uses are beginning to emerge, exemplified by NCT06932393, a not-yet-recruiting trial evaluating exosomes for hair loss treatment [126].

Although many of these trials remain early-stage or exploratory, collectively they demonstrate the diversification of exosome therapeutics beyond classical indications, expanding toward inflammatory, neurological, metabolic, and aesthetic domains. This expansion highlights both the translational promise and the ongoing regulatory and methodological challenges associated with defining optimal sources, dosing, and endpoints for exosome-based therapies.

Regulatory Hurdles

The clinical translation of exosome-based therapies faces substantial regulatory challenges arising from their complex biological nature, heterogeneous manufacturing processes, and lack of tailored guidance. The regulatory landscape for exosome-based therapeutics is still evolving, reflecting the novelty and heterogeneity of these biological nanocarriers. Both the U.S. Food and Drug Administration (FDA) and the European Medicines Agency (EMA) currently classify exosome therapeutics under existing frameworks for biological products or advanced therapy medicinal products (ATMPs), depending on their origin, manipulation level, and intended use [127]. In the United States, the FDA’s Center for Biologics Evaluation and Research (CBER), through the Office of Tissues and Advanced Therapies (OTAT), oversees clinical development of extracellular vesicle (EV)-based products, treating them similarly to human cell- and tissue-based products (HCT/Ps) that require comprehensive demonstration of safety, identity, purity, potency, and consistency under Good Manufacturing Practice (GMP) conditions. In Europe, most exosome candidates fall within the ATMP category, necessitating compliance with the EMA’s guidelines on Investigational Medicinal Products (IMPs) and centralized authorization for commercialization [128]. Up to date, no exosome-based therapy has yet achieved approval from the FDA or EMA; in the U.S., exosome products are classified as 351 biologics and must meet rigorous standards akin to those for other biologic drugs [108]. The absence of specific, globally harmonized regulatory guidelines means developers often rely on frameworks for cell and tissue products as proxies, leading to inconsistencies and ambiguity across jurisdictions [129]. Regulatory agencies consistently emphasize the need for standardized analytical characterization to define exosome identity and potency. The International Society for Extracellular Vesicles (ISEV) and the International Society for Cell & Gene Therapy (ISCT) have issued consensus guidelines (MISEV2018) describing essential physicochemical and molecular markers—including tetraspanins (CD9, CD63, CD81), TSG101, and Alix—as well as recommended assays for particle quantification, sterility, and residual nucleic acids [130]. Despite these advances, no harmonized potency assays or release criteria yet exist, representing a critical gap for both the FDA and EMA. Current efforts focus on establishing fit-for-purpose regulatory standards that align exosome product quality testing with biologics and cell-therapy benchmarks, while ensuring scalability and reproducibility of manufacturing under GMP.

In contrast, exosomes have already reached the commercial market in cosmetics and dermatology, where regulatory requirements are lighter. Companies such as BENEV (Mission Viejo, CA, USA) Company Inc., Kimera Labs (Miramar, FL, USA), and RION (Rochester, MN, USA) Aesthetics sell topical exosome-containing serums, skin rejuvenation solutions, and post-procedure products for aesthetic use. These exosomes are often derived from mesenchymal stem cells (MSCs) and marketed as cosmeceutical products that claim to enhance skin appearance without therapeutic claims, thereby avoiding the strict clinical standards required for drug approval. Although widely used in medical spas and aesthetic clinics, such products are not FDA-approved drugs, and their safety and efficacy claims are largely unverified in peer-reviewed clinical trials [120,131,132].

Overall, the convergence of regulatory initiatives and academic standardization efforts is gradually shaping a coherent framework for exosome-based therapeutics, but ambiguities remain in classification, potency evaluation, and long-term safety assessment. Addressing these issues will be essential to enable consistent regulatory approval and clinical adoption of exosome-derived medicines worldwide.

## 10. Future Directions and Emerging Trends

Exosome-based technologies have reached a pivotal point, with several therapeutic programs progressing through clinical trials. However, to fully unlock their translational potential, the field is rapidly evolving along several key technological and scientific axes. These emerging directions aim to overcome current challenges related to reproducibility, targeting, therapeutic efficacy, and scalability while expanding the scope of applications.

Exosome-Mimetic Nanoparticles and Synthetic Exosomes

To address issues of low yield and scalability, exosome-mimetic nanoparticles—artificial vesicles designed to mimic the biological composition, size, and functional properties of natural exosomes—are under investigation. These can be created via top-down approaches such as cell extrusion, or bottom-up assembly using synthetic lipids and engineered proteins. Exosome-mimetics offer advantages such as higher production yields, easier functionalization, and tunable pharmacokinetics compared to naturally derived exosomes. Several platforms are now producing hybrid vesicles that combine biological membranes with synthetic carriers, integrating exosomal targeting capabilities with the controlled payload delivery of liposomes or polymeric nanoparticles [133].

Engineered Exosomes with CRISPR, mRNA, or Immune-Modulating Cargo

Exosomes are being actively engineered as precision delivery systems for complex biologics. Recent advances have demonstrated successful exosomal delivery of CRISPR-Cas9 ribonucleoproteins, mRNA constructs, and siRNAs targeting disease-driving genes in neurodegenerative and oncologic contexts. This approach not only enhances delivery specificity but also reduces the immunogenicity and off-target risks associated with viral or synthetic carriers. Engineered exosomes carrying immune checkpoint inhibitors, tumor-associated antigens, or tolerogenic peptides are also being explored to modulate immune responses in cancer, autoimmune diseases, and transplantation [134].

Examples include the exoASO™ platform from Codiak BioSciences, which targets transcriptional regulators like STAT6 in tumor-associated macrophages [116], and Evox Therapeutics’ genome-editing exosomes, developed for disorders like Spinocerebellar Ataxia 2 and Huntington’s disease [111]. These innovations point toward a future where exosomes serve not just as delivery vehicles, but as highly programmable, multifunctional nanomedicines.

AI-Guided Design of Exosome Production and Targeting

Artificial intelligence (AI) and machine learning are increasingly being leveraged to optimize exosome development pipelines. From predicting optimal cell sources and culture conditions to identifying key surface markers for targeting, AI offers powerful tools to accelerate discovery and standardize exosome production. For instance, algorithms can be trained on high-dimensional omics data (proteomics, lipidomics, transcriptomics) to classify exosome subtypes with superior therapeutic potential or design synthetic ligands that enhance targeting efficiency [135,136]. AI is also emerging in image-based tracking and biodistribution studies, enabling non-invasive evaluation of exosome uptake, clearance, and interaction with specific tissues. As more datasets from clinical and preclinical studies become available, the role of AI in exosome drug development is expected to expand substantially, particularly in personalized medicine contexts.

Exosomes from Stem Cells or Engineered Cell Lines for Consistency

Cell source heterogeneity remains a bottleneck for reproducible exosome production. To address this, current trends favor the use of clonally engineered cell lines, such as human embryonic kidney (HEK293) cells or immortalized mesenchymal stem cells, engineered for stable exosome output, surface marker consistency, and reduced immunogenicity. Some platforms now incorporate genetic circuits to modulate exosome biogenesis, control secretion rates, or direct loading of specific cargo into the intraluminal space [137].

In parallel, stem cell-derived exosomes, particularly those from mesenchymal stem cells (MSCs), continue to be a preferred source due to their inherent anti-inflammatory and regenerative properties. Companies like EXO Biologics and Aruna Bio are advancing cGMP-compatible production systems based on MSC and neural stem cell lines, respectively, aiming for consistency in clinical-grade batches [112,119]. These efforts are expected to support standardized, scalable, and regulatory-compliant manufacturing processes across various therapeutic indications.

Combination with Stimuli-Responsive Systems

Another innovative trend is the integration of exosomes with stimuli-responsive delivery systems that enable spatiotemporal control of therapeutic release. These hybrid systems incorporate environmental triggers such as pH, temperature, enzymatic activity, redox gradients, or ultrasound to selectively release exosomal cargo in diseased tissues. For instance, tumor microenvironments often exhibit acidic pH or elevated matrix metalloproteinases, which can be used to trigger drug release from modified exosomes only within the tumor site, thereby enhancing therapeutic index and reducing systemic toxicity [138].

Additionally, exosome–hydrogel composites are being developed for localized, sustained release, particularly in regenerative medicine and wound healing. Thermo-sensitive gels, loaded with exosomes, allow for site-specific application and prolonged therapeutic activity. EverZom’s EVerGel platform exemplifies this trend, combining MSC-derived exosomes with hydrogel matrices for tissue repair in Crohn’s-related perianal fistulas [113].

Together, these emerging trends highlight a rapidly maturing field that is actively addressing its early limitations through interdisciplinary innovation. The future of exosome-based therapeutics lies not just in natural vesicles, but in rationally designed, multifunctional, and customizable delivery systems capable of addressing complex diseases. Continued integration of bioengineering, synthetic biology, and computational design, along with rigorous regulatory frameworks, will be essential for unlocking the full clinical impact of exosome science.

As novel formulations enter clinical pipelines and next-generation platforms become commercially viable, exosome systems are poised to become a cornerstone of precision nanomedicine, bridging the gap between biologics, gene therapy, and personalized drug delivery.

## 11. Conclusions

Exosome-based systems represent a compelling frontier in biomedicine, offering a unique blend of biocompatibility, intrinsic targeting, and biological functionality that sets them apart from synthetic drug carriers. Their natural capacity to traverse biological barriers, protect fragile therapeutic cargo, and modulate cellular communication underpins their immense potential across a broad range of clinical applications from regenerative medicine and oncology to neurology and gene therapy. Despite these promising attributes, significant technical and translational hurdles remain. Challenges in isolation yield, cargo loading efficiency, batch reproducibility, and regulatory clarity must be addressed through coordinated innovation in biotechnology, bioengineering, and quality control. The development of standardized protocols, robust manufacturing platforms, and well-defined potency assays will be critical to ensuring the safety, consistency, and scalability of exosome therapeutics.

Looking ahead, the field is poised for transformative progress. Emerging strategies such as synthetic exosome mimetics, engineered cargo delivery (e.g., CRISPR and mRNA), AI-driven optimization, and smart, stimuli-responsive systems offer solutions to long-standing limitations. Furthermore, the use of stem cell-derived or genetically standardized producer lines promises enhanced product consistency and regulatory compliance. As interdisciplinary collaborations continue to grow between researchers, clinicians, regulatory bodies, and industry partners, exosome-based technologies are steadily progressing from proof-of-concept models to clinically viable, precision delivery platforms. With continued scientific rigor and translational momentum, exosomes have the potential to redefine the next generation of targeted, cell-free therapies and advance the field of personalized medicine.

## Figures and Tables

**Table 1:** Comparison of ESCRT-dependent and independent pathways.

Pathway	Predominant in Cell Types	Machinery	Typical Cargo Profile	Therapeutic Relevance	Limitations
ESCRT-dependent	Epithelial, immune, many cancer cells	ESCRT-0/I/II/III, ALIX, TSG101, ubiquitin machinery	Ubiquitinated proteins, RNA-binding proteins, some miRNAs	More consistent cargo loading; easier engineering of protein/RNA sorting	Requires complex machinery; competition for ubiquitination; cell-type variability
ESCRT-independent	Fibroblasts, some tumor lines, cells with high sphingolipid metabolism	nSMase2 → ceramide, tetraspanins (CD63, CD9, CD81), lipid rafts	Lipid-enriched vesicles, signaling proteins, some RNAs	May favor membrane fusion efficiency, altered biodistribution, alternative cargo repertoire	Less predictable cargo sorting, more heterogeneity among vesicles

**Table 2:** Comparative Overview of Common Exosome Sources.

Cell Type	Key Features	Advantages	Limitations	Typical Applications
Mesenchymal Stem Cells (MSCs)	Multipotent, abundant, immune-modulatory	Low immunogenicity, regenerative potential	Heterogeneous, donor-dependent	Regenerative medicine, immunomodulation
Immune Cells (Dendritic, T, NK)	Immune signaling vesicles	Potent antigen presentation	Risk of immune activation	Cancer vaccines, immune modulation
Tumor Cells	High exosome yield, intrinsic tropism	Natural targeting to tumor microenvironment	Oncogenic content, safety concerns	Cancer biomarkers, delivery studies
Epithelial/Endothelial Cells	Structural and barrier models	High yield, relevant to vascular therapy	Limited cargo diversity	Drug delivery, vascular disease
Plant-Derived Vesicles	Naturally occurring nanovesicles	Non-toxic, scalable, oral delivery potential	Cargo characterization incomplete	Nutraceuticals, oral nanotherapy
Bacterial Vesicles (OMVs)	Derived from Gram-negative bacteria	Strong adjuvant properties	Potential immunogenicity	Vaccines, immune activation studies

**Table 3:** Comparison of isolation methods.

Method	Yield	Purity	Time	Cost	Scalability	Strengths	Limitations
Ultracentrifugation	Moderate	Moderate	Slow	Moderate	Low–Moderate	Established, large volume	Contaminants, vesicle damage
Density-gradient UC	Low–Moderate	High	Very slow	Moderate	Low	Better purity than UC	Very labor-intensive, low yield
Size-Exclusion Chromatography	Moderate	High	Moderate	Moderate	Moderate–High	Gentle, reproducible	Requires pre-concentration
Ultrafiltration	High (conc.)	Low	Fast	Low	Moderate	Rapid concentration	Shear damage, low purity
Immunoaffinity capture	Low	Very high	Moderate	High	Low	Specific subtypes isolation	Expensive, low throughput
Microfluidics-based	Low	Very high	Very fast	High	Low (currently)	Integrated diagnostic automation	Expensive, limited processing volume

**Table 4:** Comparison of loading methods for exosomes.

Method	Efficiency	Cargo Types	Exosome Integrity	Scalability	Drawbacks	Reproducibility Issues	Reference
Genetic Engineering	High	RNA, proteins	Preserved	Moderate	Labor-intensive, requires stable cell modification	Variability depends on transfection system and cell type	[48,49]
Passive Biogenesis	Low–Moderate	Small molecules	Preserved	High	Limited to lipophilic drugs	Sensitive to donor cell condition and culture method	[50]
Electroporation	Moderate (20–30%)	siRNA, miRNA	Potentially damaged	Moderate	Vesicle aggregation, altered zeta potential	Highly protocol-dependent, buffer composition critical	[51,52]
Sonication	Moderate (15–25%)	Hydrophobic drugs	Possible alteration	Moderate	Membrane disruption, loss of surface proteins	Batch-to-batch variability, sensitive to sonication intensity	[53]
Incubation	Low (<5%)	Hydrophobic drugs	Preserved	High	Very low incorporation efficiency	Dependent on drug lipophilicity, inconsistent uptake	[54,55]
Freeze–Thaw	Moderate	Proteins, peptides	Risk of aggregation	Moderate	Structural instability, loss of cargo	Inconsistent results across labs, sensitive to cycle number	[56]

**Table 6:** Comparative analysis of exosomes’ applications across therapeutic domains.

Therapeutic Domain	Main Exosome Sources	Typical Cargo	Preclinical/Clinical Status	Key Advantages	Main Challenges
Oncology	Tumor cells, MSCs, dendritic cells	Chemotherapeutics (e.g., DOX), siRNA, miRNA, proteins	Multiple preclinical models; early-phase clinical trials ongoing	Natural tumor tropism, ability to cross biological barriers	Heterogeneity, rapid clearance, immunosuppressive cargo in tumor-derived exosomes
Neurology	Neural stem cells, MSCs, engineered cells	siRNA, miRNA, neuroprotective proteins	Preclinical studies in stroke, Alzheimer’s, Parkinson’s; few early trials	Ability to cross BBB, neuroprotection, low immunogenicity	Efficient targeting and long-term safety in CNS
Infectious Diseases	Immune cells, MSCs, pathogen-infected cells	Antiviral RNAs, immunostimulatory proteins	Preclinical antiviral studies; limited clinical evaluation	Potential for targeted delivery of antiviral agents, immune activation	Scaling up antiviral exosome production, specificity
Immunology	Dendritic cells, T cells, MSCs	Antigens, immune-modulatory RNAs/proteins	Cancer vaccines tested in phase I/II trials; preclinical immune modulation	Potent antigen presentation, immune modulation capacity	Standardization of immunogenic cargo, safety, GMP compliance
Regenerative Medicine	MSCs, endothelial cells, iPSCs	Growth factors, angiogenic miRNAs, anti-apoptotic proteins	Numerous preclinical studies; several early clinical trials (wound healing, cardiac repair)	Enhances tissue regeneration, low immunogenicity, paracrine signaling	Large-scale production, cargo consistency, regulatory approval

**Table 7:** Advantages of exosomes.

Advantage	Explanation
Biocompatibility and Low Immunogenicity	Endogenous origin reduces toxicity and immune response.
Intrinsic Targeting and Homing	Surface molecules enable tissue/cell-specific uptake.
Cargo Protection and Stability	Lipid bilayer protects therapeutic payloads from degradation.
Long Circulation Time	Extended systemic half-life increases targeting probability.
Versatile Cargo Capability	Accommodates RNA, proteins, gene editing tools, small molecules.
Barrier Penetration	Able to cross BBB and other physiological barriers.
Engineering and Scalability	Amenable to surface engineering and large-scale GMP production.

**Table 8:** Limitations for the clinical translation of exosomes.

Challenge Area	Core Issues
Isolation Yield and Purity	Low protein yield per volume; contamination by non-EV particles; inconsistent isolation
Standardization	Absence of uniform protocols; variability in cell source, culture, isolation, QC
Loading Efficiency	Low encapsulation, cargo aggregation, reproducibility issues
Scalability and Regulation	Difficulty in scaling GMP; ambiguous classification; varying regulatory expectations
Immunological Risk	Potential immune activation depending on exosome source or contaminants
Stability and Storage	Poor understanding of stability, limited shelf-life, challenges in clinical-grade formulation

## Data Availability

Not applicable.

## References

[1] Serrano D.R., Kara A., Yuste I., Luciano F.C., Ongoren B., Anaya B.J., Molina G., Diez L., Ramirez B.I., Ramirez I.O. (2023). 3D Printing Technologies in Personalized Medicine, Nanomedicines, and Biopharmaceuticals. Pharmaceutics.

[2] Hadizadeh N., Bagheri D., Shamsara M., Hamblin M.R., Farmany A., Xu M., Liang Z., Razi F., Hashemi E. (2022). Extracellular vesicles biogenesis, isolation, manipulation and genetic engineering for potential in vitro and in vivo therapeutics: An overview. Front. Bioeng. Biotechnol..

[3] Di Bella M.A. (2022). Overview and Update on Extracellular Vesicles: Considerations on Exosomes and Their Application in Modern Medicine. Biology.

[4] Doyle L.M., Wang M.Z. (2019). Overview of Extracellular Vesicles, Their Origin, Composition, Purpose, and Methods for Exosome Isolation and Analysis. Cells.

[5] Shang M., Ji J.S., Song C., Gao B.J., Jin J.G., Kuo W.P., Kang H. (2017). Extracellular Vesicles: A Brief Overview and Its Role in Precision Medicine. Methods Mol. Biol..

[6] Chen J., Hu S., Liu J., Jiang H., Wang S., Yang Z. (2025). Exosomes: A double-edged sword in cancer immunotherapy. MedComm.

[7] Zhang X., Lu Y., Wu S., Zhang S., Li S., Tan J. (2022). An Overview of Current Research on Mesenchymal Stem Cell-Derived Extracellular Vesicles: A Bibliometric Analysis From 2009 to 2021. Front. Bioeng. Biotechnol..

[8] Li L., Yang Z., Li J. (2024). Exosomes and SARS-CoV-2 infection. Front. Immunol..

[9] Lim C., Blocher McTigue W.C. (2024). Form Equals Function: Influence of Coacervate Architecture on Drug Delivery Applications. ACS Biomater. Sci. Eng..

[10] Souri M., Yim W., Halder M., Jin Z., Jokerst J.V. (2025). Coacervate-Based Delivery Systems: Bridging Fundamentals and Applications. ACS Appl. Mater. Interfaces.

[11] Chen H., Ma M., Zhang L., Wang Z., Sun H., Liu C., Zhang L., Zhang W. (2025). An effective strategy based on electrostatic interaction for the simultaneous sequential purification and isolation of exosomes. Mikrochim. Acta.

[12] Colao I.L., Corteling R., Bracewell D., Wall I. (2018). Manufacturing Exosomes: A Promising Therapeutic Platform. Trends Mol. Med..

[13] Yu J., Sane S., Kim J.E., Yun S., Kim H.J., Jo K.B., Wright J.P., Khoshdoozmasouleh N., Lee K., Oh H.T. (2023). Biogenesis and delivery of extracellular vesicles: Harnessing the power of EVs for diagnostics and therapeutics. Front. Mol. Biosci..

[14] Gurung S., Perocheau D., Touramanidou L., Baruteau J. (2021). The exosome journey: From biogenesis to uptake and intracellular signalling. Cell Commun. Signal.

[15] van Niel G., D’Angelo G., Raposo G. (2018). Shedding light on the cell biology of extracellular vesicles. Nat. Rev. Mol. Cell Biol..

[16] Jin Y., Ma L., Zhang W., Yang W., Feng Q., Wang H. (2022). Extracellular signals regulate the biogenesis of extracellular vesicles. Biol. Res..

[17] Xu K., Feng H., Zhao R., Huang Y. (2025). Targeting Tetraspanins at Cell Interfaces: Functional Modulation and Exosome-Based Drug Delivery for Precise Disease Treatment. ChemMedChem.

[18] Moeinzadeh L., Razeghian-Jahromi I., Zarei-Behjani Z., Bagheri Z., Razmkhah M. (2022). Composition, Biogenesis, and Role of Exosomes in Tumor Development. Stem Cells Int..

[19] Isaac R., Castellani F., Ynig W., Olefsky J.M. (2021). Exosomes as mediators of intercellular crosstalk in metabolism. Cell Metab..

[20] Bischoff J.P., Schulz A., Morrison H. (2022). The role of exosomes in intercellular and inter-organ communication of the peripheral nervous system. FEBS Lett..

[21] Su S.A., Xie Y., Fu Z., Wang Y., Wang J.A., Xiang M. (2017). Emerging role of exosome-mediated intercellular communication in vascular remodeling. Oncotarget.

[22] van Niel G., Carter D.R.F., Clayton A., Lambert D.W., Raposo G., Vader P. (2022). Challenges and directions in studying cell-cell communication by extracellular vesicles. Nat. Rev. Mol. Cell Biol..

[23] Hao Q., Wu Y., Wu Y., Wang P., Vadgama J.V. (2022). Tumor-Derived Exosomes in Tumor-Induced Immune Suppression. Int. J. Mol. Sci..

[24] Poggio M., Hu T., Pai C.C., Chu B., Belair C.D., Chang A., Montabana E., Lang U.E., Fu Q., Fong L. (2019). Suppression of Exosomal PD-L1 Induces Systemic Anti-tumor Immunity and Memory. Cell.

[25] Liu J., Peng X., Yang S., Li X., Huang M., Wei S., Zhang S., He G., Zheng H., Fan Q. (2022). Extracellular vesicle PD-L1 in reshaping tumor immune microenvironment: Biological function and potential therapy strategies. Cell Commun. Signal.

[26] Tang Y., Zhou Y., Li H.J. (2021). Advances in mesenchymal stem cell exosomes: A review. Stem Cell Res. Ther..

[27] Lotfy A., AboQuella N.M., Wang H. (2023). Mesenchymal stromal/stem cell (MSC)-derived exosomes in clinical trials. Stem Cell Res. Ther..

[28] Marote A., Teixeira F.G., Mendes-Pinheiro B., Salgado A.J. (2016). MSCs-Derived Exosomes: Cell-Secreted Nanovesicles with Regenerative Potential. Front. Pharmacol..

[29] Luo S., Chen J., Xu F., Chen H., Li Y., Li W. (2023). Dendritic Cell-Derived Exosomes in Cancer Immunotherapy. Pharmaceutics.

[30] Whitesie T.L. (2016). Tumor-Derived Exosomes and Their Role in Cancer Progression. Adv. Clin.Chem..

[31] Ren Y., Zhang H. (2023). Emerging role of exosomes in vascular diseases. Front. Cardiovasc. Med..

[32] Mu J., Zhuang X., Wang Q., Jiang H., Deng Z.-B., Wang B., Zhang L., Kakar S.S., Jun Y., Miller D. (2014). Interspecies communication between plant and mouse gut host cells through edible plant derived exosome-like nanoparticles. Mol. Nutr. Food Res..

[33] Gerritzen M.J.H., Martens D.E., Wijffels R.H., van der Pol L., Stork M. (2017). Bioengineering bacterial outer membrane vesicles as vaccine platform. Biotechnol. Adv..

[34] Gao J., Li A., Hu J., Feng L., Liu L., Shen Z. (2022). Recent developments in isolating methods for exosomes. Front. Bioeng. Biotechnol..

[35] Sidhom K., Obi P.O., Saleem A. (2020). A Review of Exosomal Isolation Methods: Is Size Exclusion Chromatography the Best Option?. Int. J. Mol. Sci..

[36] Wu Y., Wang Y., Lu Y., Luo X., Huang Y., Xie T., Pilarsky C., Dang Y., Zhang J. (2022). Microfluidic Technology for the Isolation and Analysis of Exosomes. Micromachines.

[37] Lai J.J., Chau Z.L., Chen S.Y., Hill J.J., Korpany K.V., Liang N.W., Lin L.H., Lin Y.H., Liu J.K., Liu Y.C. (2022). Exosome Processing and Characterization Approaches for Research and Technology Development. Adv. Sci..

[38] Johnsen K.B., Gudbergsson J.M., Skov M.N., Christiansen G., Gurevich L., Moos T., Duroux M. (2016). Evaluation of electroporation-induced adverse effects on adipose-derived stem cell exosomes. Cytotechnology.

[39] Hoshino A., Costa-Silva B., Shen T.L., Rodrigues G., Hashimoto A., Tesic Mark M., Molina H., Kohsaka S., Di Giannatale A., Ceder S. (2015). Tumour exosome integrins determine organotropic metastasis. Nature.

[40] Liam-Or R., Faruqu F.N., Walters A., Han S., Xu L., Wang J.T., Oberlaender J., Sanchez-Fueyo A., Lombardi G., Dazzi F. (2024). Cellular uptake and in vivo distribution of mesenchymal-stem-cell-derived extracellular vesicles are protein corona dependent. Nat. Nanotechnol..

[41] Yi Y.W., Lee J.H., Kim S.Y., Pack C.G., Ha D.H., Park S.R., Youn J., Cho B.S. (2020). Advances in Analysis of Biodistribution of Exosomes by Molecular Imaging. Int. J. Mol. Sci..

[42] Lau S.Y., Kang M., Hisey C.L., Chamley L.W. (2023). Studying exogenous extracellular vesicle biodistribution by in vivo fluorescence microscopy. Dis. Model. Mech..

[43] Majka M., Durak-Kozica M., Kaminska A., Opalinska A., Szcech M., Stepien E. (2017). The effects of subdiffusion on the NTA size measurements of extracellular vesicles in biological samples. arXiv.

[44] Woud W.W., van der Pol E., Mul E., Hoogduijn M.J., Baan C.C., Boer K., Merino A. (2022). An imaging flow cytometry-based methodology for the analysis of single extracellular vesicles in unprocessed human plasma. Commun. Biol..

[45] Gurunathan S., Kang M.H., Jeyaraj M., Qasim M., Kim J.H. (2019). Review of the Isolation, Characterization, Biological Function, and Multifarious Therapeutic Approaches of Exosomes. Cells.

[46] Vader P., Mol E.A., Pasterkamp G., Schiffelers R.M. (2016). Extracellular vesicles for drug delivery. Adv. Drug Deliv. Rev..

[47] Hung M.E., Leonard J.N. (2016). A platform for actively loading cargo RNA to elucidate limiting steps in EV-mediated delivery. J. Extracell. Vesicles.

[48] Alvarez-Erviti L., Seow Y., Yin H., Betts C., Lakhal S., Wood M.J. (2011). Delivery of siRNA to the mouse brain by systemic injection of targeted exosomes. Nat. Biotechnol..

[49] Kojima R., Bojar D., Rizzi G., Hamri G.C., El-Baba M.D., Saxena P., Auslander S., Tan K.R., Fussenegger M. (2018). Designer exosomes produced by implanted cells intracerebrally deliver therapeutic cargo for Parkinson’s disease treatment. Nat. Commun..

[50] Pascucci L., Cocce V., Bonomi A., Ami D., Ceccarelli P., Ciusani E., Vigano L., Locatello A., Sisto F., Doglia S.M. (2014). Paclitaxel is incorporated by mesenchymal stromal cells and released in exosomes that inhibit in vitro tumor growth: A new approach for drug delivery. J. Control Release.

[51] Wahlgren J., Karlson T., Brisslert M., Sani F.V., Telemo E., Sunnerhagen P., Valadi H. (2012). Plasma exosomes can deliver exogenous short interfering RNA to monocytes and lymphocytes. Nucleic Acids Res..

[52] Kooijmans S.A.A., Stremersch S., Braeckmans K., de Smedt S.C., Hendrix A., Wood M.J.A., Schiffelers R.M., Raemdonck K., Vader P. (2013). Electroporation-induced siRNA precipitation obscures the efficiency of siRNA loading into extracellular vesicles. J. Control Release.

[53] Haney M.J., Klyanchko N.L., Zhao Y., Gupta R., Plotnikova E.G., He Z., Patel T., Piroyan A., Sokolsky M., Kabanov A.V. (2015). Exosomes as drug delivery vehicles for Parkinson’s disease therapy. J. Control Release.

[54] Tian Y., Li S., Song J., Ji T., Zhu M., Anderson G.J., Wei J., Nie G. (2014). A doxorubicin delivery platform using engineered natural membrane vesicle exosomes for targeted tumor therapy. Biomaterials.

[55] Sun D., Zhuang X., Xiang X., Liu Y., Zhang S., Liu C., Barnes S., Grizzle W., Miller D., Zhang H.G. (2010). A novel nanoparticle drug delivery system: The anti-inflammatory activity of curcumin is enhanced when encapsulated in exosomes. Mol. Ther..

[56] Cheng Y., Zeng Q., Han Q., Xia W. (2019). Effect of pH, temperature and freezing-thawing on quantity changes and cellular uptake of exosomes. Protein Cell.

[57] Gabaran S.G., Ghasemzadeh N., Rahnama M., Karatas E., Akbari A., Rezaie J. (2025). Functionalized exosomes for targeted therapy in cancer and regenerative medicine: Genetic, chemical, and physical modifications. Cell Commun. Signal.

[58] Johnson V., Vasu S., Kumar U.S., Kumar M. (2023). Surface-Engineered Extracellular Vesicles in Cancer Immunotherapy. Cancers.

[59] Li J., Wang J., Chen Z. (2025). Emerging role of exosomes in cancer therapy: Progress and challenges. Mol. Cancer.

[60] Javid H., Oryani M.A., Rezagholinejad N., Esparham A., Tajaldini M., Karimi-Shahri M. (2024). RGD peptide in cancer targeting: Benefits, challenges, solutions, and possible integrin-RGD interactions. Cancer Med..

[61] Wei Z., Zhou Y., Wang R., Wang J., Chen Z. (2022). Aptamers as Smart Ligands for Targeted Drug Delivery in Cancer Therapy. Pharmaceutics.

[62] Liu Q., Li D., Pan X., Liang Y. (2023). Targeted therapy using engineered extracellular vesicles: Principles and strategies for membrane modification. J. Nanobiotechnol..

[63] Choi H., Choi Y., Yim H.Y., Mirzaaghasi A., Yoo J.K., Choi C. (2021). Biodistribution of Exosomes and Engineering Strategies for Targeted Delivery of Therapeutic Exosomes. Tissue Eng. Regen. Med..

[64] Parada N., Romero-Trujillo A., Georges N., Alcayaga-Miranda F. (2021). Camouflage strategies for therapeutic exosomes evasion from phagocytosis. J. Adv. Res..

[65] Palakurthi S.S., Shah B., Kapre S., Charbe N., Immanuel S., Pasham S., Thalla M., Jain A., Palakurthi S. (2024). A comprehensive review of challenges and advances in exosome-based drug delivery systems. Nanoscale Adv..

[66] Ren L., Zhang D., Pang L., Liu S. (2024). Extracellular vesicles for cancer therapy: Potential, progress, and clinical challenges. Front. Bioeng. Biotechnol..

[67] Li L., Wang F., Zhu D., Hu S., Cheng K., Li Z. (2025). Engineering exosomes and exosome-like nanovesicles for improving tissue targeting and retention. Fundam. Res..

[68] Attar F.A., Irani S., Oloomi M., Bolhassani A., Geranpayeh L., Atyabi F. (2025). Doxorubicin loaded exosomes inhibit cancer-associated fibroblasts growth: In vitro and in vivo study. Cancer Cell Int..

[69] Raguraman R., Bhavsar D., Kim D., Ren X., Sikavitsas V., Munshi A., Ramesh R. (2023). Tumor-targeted exosomes for delivery of anticancer drugs. Cancer Lett..

[70] Xu W., Wang K., Wang K., Zhao Y., Yang Z., Li X. (2024). Key Magnetized Exosomes for Effective Targeted Delivery of Doxorubicin Against Breast Cancer Cell Types in Mice Model. Int. J. Nanomed..

[71] Zhang W., Jiang X., Bao J., Wang Y., Liu H., Tang L. (2018). Exosomes in Pathogen Infections: A Bridge to Deliver Molecules and Link Functions. Front. Immunol..

[72] Peng Y., Yang Y., Li Y., Shi T., Luan Y., Yin C. (2023). Exosome and virus infection. Front. Immunol..

[73] Schorey J.S., Harding C.V. (2016). Extracellular vesicles and infectious diseases: New complexity to an old story. J. Clin. Investig..

[74] Gorgzadeh A., Nazari A., Ali Ehsan Ismaeel A., Safarzadeh D., Hassan J.A.K., Mohammadzadehsaliani S., Kheradjoo H., Yasamineh P., Yasamineh S. (2024). A state-of-the-art review of the recent advances in exosome isolation and detection methods in viral infection. Virol. J..

[75] Teymouri S., Pourhajibagher M., Bahador A. (2024). Exosomes: Friends or Foes in Microbial Infections?. Infect. Disord. Drug Targets.

[76] Serrano Lopez D.R., Lalatsa A. (2013). Peptide pills for brain diseases? Reality and future perspectives. Ther. Deliv..

[77] Chen L., Xiong Y., Chopp M., Zhang Y. (2019). Engineered exosomes enriched with select microRNAs amplify their therapeutic efficacy for traumatic brain injury and stroke. Front. Cell. Neurosci..

[78] Fatima S., Qaiser A., Andleeb S., Hashmi A.H., Manzoor S. (2023). Navigating the brain: The role of exosomal shuttles in precision therapeutics. Front. Neurol..

[79] Sanadgol N., Abedi M., Hashemzaei M., Kamran Z., Khalseh R., Beyer C., Voelz C. (2025). Exosomes as nanocarriers for brain-targeted delivery of therapeutic nucleic acids: Advances and challenges. J. Nanobiotechnol..

[80] Abdelsalam M., Ahmed M., Osaid Z., Hamoudi R., Harati R. (2023). Insights into Exosome Transport through the Blood–Brain Barrier and the Potential Therapeutical Applications in Brain Diseases. Pharmaceuticals.

[81] Jain S., Murmu A., Chauhan A. (2025). Advancing Alzheimer’s disease therapy through engineered exosomal Macromolecules. Brain Res..

[82] Sun M., Chen Z. (2024). Unveiling the Complex Role of Exosomes in Alzheimer’s Disease. J. Inflamm. Res..

[83] Zubair M., Abouelnzar F.A., Iqbal M.A., Pan J., Zheng X., Chen T., Shen W., Yin J., Yongmin Y., Lui P. (2025). Mesenchymal stem cell-derived exosomes as a plausible immunomodulatory therapeutic tool for inflammatory diseases. Front. Cell Dev. Biol..

[84] Barbetta C., Bonomi F., Lepri G., Furst D.E., Randone S.B., Guiducci S. (2025). Mesenchymal Stem-Cell-Derived Exosomes and MicroRNAs: Advancing Cell-Free Therapy in Systemic Sclerosis. Cells.

[85] Song D., He C., Ocansey D.K.W., Wang B., Wu Y., Mao F. (2025). Mesenchymal stem cell in immunomodulation of dendritic cells: Implications for inflammatory bowel disease therapy. Autoimmun. Rev..

[86] Mignini I., Piccirilli G., Termite F., Paratore M., Esposto G., Laterza L., Scaldaferri F., Ainora M.E., Gasbarrini A., Zocco M.A. (2023). Extracellular Vesicles: Novel Potential Therapeutic Agents in Inflammatory Bowel Diseases. Cells.

[87] Wan T., Zhong J., Pan Q., Zhou T., Ping Y., Liu X. (2022). Exosome-mediated delivery of Cas9 ribonucleoprotein complexes for tissue-specific gene therapy of liver diseases. Sci. Adv..

[88] Gee P., Lung M.S.Y., Okuzaki Y., Sasakawa N., Iguchi T., Makita Y., Hozumi H., Miura Y., Yang L.F., Iwasaki M. (2020). Extracellular nanovesicles for packaging of CRISPR-Cas9 protein and sgRNA to induce therapeutic exon skipping. Nat. Commun..

[89] Bhavanisha Rithiga S., Dhar R., Devi A. (2025). Exosomes-mediated CRISPR/Cas delivery: A cutting-edge frontier in cancer gene therapy. Gene.

[90] Lai R.C., Arslan F., Lee M.M., Sze N.S., Choo A., Chen T.S., Salto-Tellez M., Timmers L., Lee C.N., El Oakley R.M. (2010). Exosome secreted by MSC reduces myocardial ischemia/reperfusion injury. Stem Cell Res..

[91] Shabbir A., Cox A., Rodriguez-Menocal L., Salgado M., Van Badiavas E. (2015). Mesenchymal Stem Cell Exosomes Induce Proliferation and Migration of Normal and Chronic Wound Fibroblasts, and Enhance Angiogenesis In Vitro. Stem Cells Dev..

[92] Jiang K., Jiang T., Chen Y., Mao X. (2021). Mesenchymal Stem Cell-Derived Exosomes Modulate Chondrocyte Glutamine Metabolism to Alleviate Osteoarthritis Progression. Mediat. Inflamm..

[93] Zhong D., Cao Y., Li C.J., Li M., Rong Z.J., Jiang L., Guo Z., Lu H.B., Hu J.Z. (2020). Neural stem cell-derived exosomes facilitate spinal cord functional recovery after injury by promoting angiogenesis. Exp. Biol. Med..

[94] Wang Y., Shen X., Song S., Chen Y., Wang Y., Liao J., Chen N., Zeng L. (2023). Mesenchymal stem cell-derived exosomes and skin photoaging: From basic research to practical application. Photodermatol. Photoimmunol. Photomed..

[95] Tienda-Vazquez M.A., Hanel J.M., Marquez-Arteaga E.M., Salgado-Alvarez A.P., Scheckhuber C.Q., Alanis-Gomez J.R., Espinoza-Silva J.I., Ramos-Kuri M., Hernandez-Rosas F., Melchor-Martinez E.M. (2023). Exosomes: A Promising Strategy for Repair, Regeneration and Treatment of Skin Disorders. Cells.

[96] Li X., Zhang D., Yu Y., Wang L., Zhao M. (2024). Umbilical cord-derived mesenchymal stem cell secretome promotes skin regeneration and rejuvenation: From mechanism to therapeutics. Cell Prolif..

[97] Kim H., Jang H., Cho H., Choi J., Hwang K.Y., Choi Y., Kim S.H., Yang Y. (2021). Recent Advances in Exosome-Based Drug Delivery for Cancer Therapy. Cancers.

[98] Kim H.I., Park J., Zhu Y., Wang X., Han Y., Zhang D. (2024). Recent advances in extracellular vesicles for therapeutic cargo delivery. Exp. Mol. Med..

[99] He J., Ren W., Wang W., Han W., Jiang L., Zhang D., Guo M. (2022). Exosomal targeting and its potential clinical application. Drug Deliv. Transl. Res..

[100] Zeng H., Guo S., Ren X., Wu Z., Liu S., Yao X. (2023). Current Strategies for Exosome Cargo Loading and Targeting Delivery. Cells.

[101] Dimik M., Abeysinghe P., Logan J., Mitchell M. (2023). The exosome: A review of current therapeutic roles and capabilities in human reproduction. Drug Deliv. Transl. Res..

[102] Dilsiz N. (2024). A comprehensive review on recent advances in exosome isolation and characterization: Toward clinical applications. Transl. Oncol..

[103] Rezaie J., Feghhi M., Etemadi T. (2022). A review on exosomes application in clinical trials: Perspective, questions, and challenges. Cell Commun. Signal.

[104] Chen J., Li P., Zhang T., Xu Z., Huang X., Wang R., Du L. (2021). Review on Strategies and Technologies for Exosome Isolation and Purification. Front. Bioeng. Biotechnol..

[105] Rankin-Turner S., Vader P., O’Driscoll L., Giebel B., Heaney L.M., Davies O.G. (2021). A call for the standardised reporting of factors affecting the exogenous loading of extracellular vesicles with therapeutic cargos. Adv. Drug Deliv. Rev..

[106] Davies O., Rafiq Q. (2017). Considerations for the bioprocessing, manufacture and translation of extracellular vesicles for therapeutic applications. Cell Gene Ther. Insights.

[107] Verma N., Arora S. (2025). Navigating the Global Regulatory Landscape for Exosome-Based Therapeutics: Challenges, Strategies, and Future Directions. Pharmaceutics.

[108] Thakur A., Rai D. (2024). Global requirements for manufacturing and validation of clinical grade extracellular vesicles. J. Liq. Biopsy.

[109] Aegle Therapeutics. https://aegletherapeutics.com/ev-therapy/.

[110] Capricor Tehrapeutics. https://www.capricor.com/our-science.

[111] Evox Therapeutics. https://www.evoxtherapeutics.com/pipeline/.

[112] Aruna Bio. https://www.arunabio.com/pipeline.

[113] Everzom. https://everzom.com/innovation-platform/mother-cell-source/.

[114] Aposcience. https://www.aposcience.at/company/.

[115] Codiak Bioscience. https://www.biospace.com/employer/1935177/codiak-biosciences.

[116] Codiak Bioscience Programme. https://www.biospace.com/codiak-presents-preclinical-data-on-exoaso-stat6-and-exoaso-c-ebp%CE%B2-programs-at-the-society-for-immunotherapy-of-cancer-sitc-2022-annual-meeting.

[117] Exoexpert. https://exoxpert.com/exoxpert-an-exosome-cdmo-launched-by-exo-biologics/?utm_source=chatgpt.com.

[118] Exoexpert MSC-Derived Extracellular Vesicles. https://exoxpert.com/research-paper-evs-from-ms-umbilical-cord-cells-exert-protection-against-oxidative-stress-and-fibrosis-in-a-rat-model-of-bpd/?utm_source=chatgpt.com.

[119] EXO Biologics and ExoXpert Reach Two Critical Milestones that Advance Exosomes. https://www.biopharminternational.com/view/exo-biologics-exoxpert-reach-two-critical-milestones-advance-exosomes?utm_source=chatgpt.com.

[120] Kinera Labs Vive. https://kimeravive.com/.

[121] Exosomas Exocobio. https://dermatologiaestoril.cl/products/exosomas-exocobio.

[122] Rion. https://riontx.com/pipeline/.

[123] SCTBio. https://www.sctbio.com/.

[124] Lonza. https://www.lonza.com/specialized-modalities/cell-and-gene/exosomes.

[125] RoosterBIo. https://www.roosterbio.com/products/quickship-hmsc-exosomes-extracellular-vesicles-evs/?gad_source=1&gad_campaignid=16975550564&gbraid=0AAAAABLy-paHChATjcqfBX1vXWpyuf78U&gclid=Cj0KCQjwtMHEBhC-ARIsABua5iQtpqJKfz0aM_qbgbzGmQCAGTkv7NZTL5VNfURxu2h0xfroHxGZMDEaAhZ2EALw_wcB.

[126] Clinical Trials Using Exosomes. https://clinicaltrials.gov/.

[127] Li Q., Li Y., Shao J., Sun J., Hu L., Yun X., Liuqing C., Gong L., Wu S. (2025). Exploring Regulatory Frameworks for Exosome Therapy: Insights and Perspectives. Health Care Sci..

[128] Wang C.-K., Tsai T.-H., Lee C.-H. (2024). Regulation of exosomes as biologic medicines: Regulatory challenges faced in exosome development and manufacturing processes. Clin. Transl. Sci..

[129] Unravelling the Regulatory Riddle for Exosome-Based Therapies. https://www.biopharma-excellence.com/2020-7-3-unravelling-the-regulatory-riddle-for-exosome-based-therapies/?utm_source=chatgpt.com.

[130] Humbert C., Cordier C., Drut I., Hamrick M., Wong J., Bellamy V., Flaire J., Bakshy K., Dingli F., Loew D. (2025). GMP-Compliant Process for the Manufacturing of an Extracellular Vesicles-Enriched Secretome Product Derived From Cardiovascular Progenitor Cells Suitable for a Phase I Clinical Trial. J. Extracell. Vesicles.

[131] Rion Aesthetics. https://www.rionaesthetics.com/.

[132] Benev. https://www.benev.com/.

[133] Li Y.J., Wu J.Y., Liu J., Xu W., Qiu X., Huang S., Hu X.B., Xiang D.X. (2021). Artificial exosomes for translational nanomedicine. J. Nanobiotechnol..

[134] Lu Y., Godbout K., Lamothe G., Tremblay J.P. (2023). CRISPR-Cas9 delivery strategies with engineered extracellular vesicles. Mol. Ther. Nucleic Acids.

[135] Serrano D.R., Luciano F.C., Anaya B.J., Ongoren B., Kara A., Molina G., Ramirez B.I., Sanchez-Guirales S.A., Simon J.A., Tomietto G. (2024). Artificial Intelligence (AI) Applications in Drug Discovery and Drug Delivery: Revolutionizing Personalized Medicine. Pharmaceutics.

[136] Baghban N., Kodam S.P., Ullah M. (2023). Role of CD9 Sensing, AI, and Exosomes in Cellular Communication of Cancer. Int. J. Stem Cell Res. Ther..

[137] Schwarz G., Ren X., Xie W., Guo H., Jiang Y., Zhang J. (2025). Engineered exosomes: A promising drug delivery platform with therapeutic potential. Front. Mol. Biosci..

[138] Lee Y.J., Shin K.J., Chae Y.C. (2024). Regulation of cargo selection in exosome biogenesis and its biomedical applications in cancer. Exp. Mol. Med..

